# Phosphate deficiency reduces nodule formation through a phosphate starvation response-like protein in *Phaseolus vulgaris*

**DOI:** 10.1093/pcp/pcaf069

**Published:** 2025-06-26

**Authors:** Jawahar Singh, Ana Belén Mendoza-Soto, Manish Tiwari, Tomas Tonaltsintle Acevedo-Sandoval, Damien Formey, Jean-Michel Ané, Mariel C Isidra-Arellano, Oswaldo Valdés-López

**Affiliations:** Laboratorio de Genómica Funcional de Leguminosas, Departamento de Biología, Facultad de Estudios Superiores Iztacala, Universidad Nacional Autónoma de México, Tlalnepantla 54090, Mexico; Sainsbury Laboratory, University of Cambridge, Cambridge, CB2 ILR, UK; Laboratorio de Genómica Funcional de Leguminosas, Departamento de Biología, Facultad de Estudios Superiores Iztacala, Universidad Nacional Autónoma de México, Tlalnepantla 54090, Mexico; Department of Bacteriology, University of Wisconsin, Microbial Science Building, 1550 Linden Drive, Madison, WI 53706, USA; Laboratorio de Genómica Funcional de Leguminosas, Departamento de Biología, Facultad de Estudios Superiores Iztacala, Universidad Nacional Autónoma de México, Tlalnepantla 54090, Mexico; Centro de Ciencias Genómicas, Programa de Genómica Funcional de Eucariotes, Universidad Nacional Autónoma de México, Cuernavaca, Morelos 62210, Mexico; Department of Bacteriology, University of Wisconsin, Microbial Science Building, 1550 Linden Drive, Madison, WI 53706, USA; Department of Agronomy, University of Wisconsin, 1575 Linden Drive, Madison, WI 53706, USA; Laboratorio de Genómica Funcional de Leguminosas, Departamento de Biología, Facultad de Estudios Superiores Iztacala, Universidad Nacional Autónoma de México, Tlalnepantla 54090, Mexico; Laboratorio de Genómica Funcional de Leguminosas, Departamento de Biología, Facultad de Estudios Superiores Iztacala, Universidad Nacional Autónoma de México, Tlalnepantla 54090, Mexico

**Keywords:** root nodule symbiosis, transcription factors, autoregulation of nodulation, nodule inception, Too Much Love, nodulation autoregulation receptor kinase

## Abstract

Phosphate deficiency reduces nodule formation in various legumes, which hinders nitrogen fixation and crop yield. We previously showed that phosphate deficiency reduces nodule formation by activating the autoregulation of nodulation (AON) pathway. We also observed that some genetic components of the AON pathway contain Phosphate Starvation Response 1 binding site *cis*-regulatory elements in their promoter regions, which are recognized by the Phosphate Starvation Response 1 transcription factor. This evidence led us to hypothesize that host plant phosphate levels regulate the expression of genes essential for forming nodules through a PHR-Like protein. In the present study, we provide evidence supporting the participation of PvPHR-Like 7 (PvPHR-L7) in regulating nodule formation in *Phaseolus vulgaris*. Modulation of *PvPHR-L7’s* expression by RNA interference and overexpression suggested that this transcription factor may control the expression of crucial symbiotic genes involved in nodule development in *P. vulgaris*. An RT-qPCR analysis revealed that the expression of *PvPHR-L7*, *PvNIN*, and *PvTML* is regulated in accordingly to the plant host Pi levels. Transactivation assays in *Nicotiana benthamiana* and *P. vulgaris* transgenic roots indicate that PvPHR-L7 can upregulate the expression of *PvNIN* and *PvTML* in the absence of rhizobia. In contrast, PvPHR-L7 downregulates the expression of *PvNIN* under symbiotic conditions with rhizobia. The data presented shed light on the potential role that PvPHR-L7 plays in the root nodule symbiosis.

## Introduction

The availability of nutrients in the soil is a significant factor determining plant growth and productivity. Among many nutrients, nitrogen and phosphorus are two of the most important, as they are essential building blocks for a wide range of biomolecules. Plants primarily uptake nitrogen from the soil as nitrate, whereas phosphorus is exclusively absorbed as inorganic phosphate (Pi) (Zhong et al. 2023). However, nitrate and Pi are scarce in the soil, negatively impacting plant growth and development.

Synthetic fertilizers are widely used to increase crop yield, but overuse harms soil and water bodies. Root endosymbiosis is a sustainable strategy that can reduce our dependence on synthetic fertilizers (Singh et al. 2023). For instance, legumes are associated with nitrogen-fixing bacteria collectively known as rhizobia. Legumes host rhizobia in specialized root organs called nodules, in which rhizobia fix the atmospheric nitrogen (Roy et al. 2020). Legumes obtain fixed nitrogen (i.e. ammonium) through this symbiosis, significantly enhancing their growth in nitrogen-deficient soils and serving as a natural biofertilizer (Zahran 1999). Legumes interact symbiotically with arbuscular mycorrhizal (AM) fungi like other land plants. Through this symbiosis, legumes obtain different mineral nutrients, the most relevant being phosphorus (Wang et al. 2018).

The first step in establishing a successful symbiosis between legumes and rhizobia begins with recognizing rhizobia. Legumes release flavonoid compounds to the rhizosphere, where compatible rhizobia detect them (Roy et al. 2020). In turn, rhizobia produce lipo-chitooligosaccharides (LCOs), also known as Nod factors (NFs), which the legume host perceives through LysM receptor-like kinases (Limpens et al. 2003, Madsen et al. 2003, Radutoiu et al. 2003, Arrighi et al. 2006). Rhizobial-derived LCOs perception activates the common symbiosis signaling pathway, which controls the establishment of both root nodule- and AM symbiosis (Roy et al. 2020). One of the critical components of this pathway is the rapid and sustained oscillation of the nuclear and perinuclear calcium concentrations, known as calcium spiking (Ehrhardt et al. 1996, Kanamori et al. 2006, Peiter et al. 2007, Saito et al. 2007, Charpentier et al. 2016). The decoding of these calcium signatures by a calcium/calmodulin-dependent protein kinase (CCaMK) activates the transcription of a set of transcription factor-encoding genes, including *Nodule Inception* (*NIN*) (Singh and Parniske 2012, Singh et al. 2014). NIN controls different stages of root nodule symbiosis, from root hair curling required for infection to organogenesis and symbiont regulation (Schauster et al. 1999, Marsh et al. 2007, Liu et al. 2019, Feng et al. 2021).

Symbiotic nitrogen fixation is an energy-demanding process that requires many plant-derived carbon sources (Ferguson et al. 2019). Legumes tightly regulate the number of nodules to avoid an energetic imbalance through an intricate long-distance signaling pathway called autoregulation of nodulation (AON) (Ferguson et al. 2019). This process begins with the production of root-derived CLAVATA3/EMBRYO SURROUNDING REGION-RELATED (CLE) peptides called Rhizobia Induced CLE peptide 1 (RIC1) and RIC2 in *Phaseolus vulgaris* and soybean (Reid et al. 2011, Ferguson et al. 2014). The production of these two CLE peptides begins with the first rhizobia-induced cortical cell division and continues through nodule development and symbiotic nitrogen fixation (Lim et al. 2011, Yoro et al. 2019). NIN regulates the activation of their transcription in *Lotus japonicus* or *Medicago truncatula* (Soyano et al. 2014, Laffont et al. 2020). The mature RIC1 and RIC2 peptides are transported via the xylem from the root to the shoot, where they are detected by the nodulation autoregulation receptor kinase (NARK) (Krusell et al. 2002, Searle et al. 2003, Schnabel et al. 2005, Ferguson et al. 2014). The CLE peptide perception activates a signaling pathway that produces shoot-derived signals, which are transported to the root to inhibit further nodule development (Ferguson et al. 2019). Accordingly, the Kelch-repeat-containing F-box protein Too Much Love (TML) and the miRNA miR2111 regulate the number of nodules (Takahara et al. 2013, Tsikou et al. 2018). The *nark* and *tml* mutant and transgenic plants overexpressing *miRNA2111* show a super-nodulation phenotype, confirming that these genetic components are crucial in maintaining an optimal number of nodules (Krusell et al. 2002, Searle et al. 2003, Schnabel et al. 2005, Takahara et al. 2013, Tsikou et al. 2018).

Most agricultural soils around the world have sub-optimal levels of Pi. Plants have developed a Pi starvation response (PSR) that adapts to Pi-deficient conditions and fulfills their nutritional needs (Isidra-Arellano et al. 2021). The PSR system is tightly regulated by Phosphate Starvation Response 1 (PHR1) and PHR-like (PHL) proteins, which belong to the Myeloblastoma-coiled-coil (Myb-CC) transcription factor family (Rubio et al. 2001, Bustos et al. 2010). PHR1 and PHL bind to the Phosphate Starvation Response 1 binding site (P1BS) *cis*-regulatory element, a partially palindromic sequence motif (GNATATNC) frequently found in the promoter regions of PSR-associated genes (Rubio et al. 2001). PHR1 and PHL modulate the expression of various Pi starvation-responsive genes participating in the Pi uptake, translocation, and homeostasis (Rubio et al. 2001, Isidra-Arellano et al. 2021).

PHR2 (the orthologous of PHR1 in rice) also regulates endosymbiosis with AM fungi (Shi et al. 2021, Das et al. 2022). PHR2 directly targets several genes involved in AM symbiosis that contain the P1BS element in their promoter regions (Shi et al. 2021, Das et al. 2022). Among the targeted genes are those involved in strigolactone biosynthesis and LysM-Receptor-Like kinases responsible for pre-contact signaling (Shi et al. 2021, Das et al. 2022). Additionally, genes belonging to the common symbiosis signaling pathway (i.e. Symbiosis Receptor Kinase: SYMRK, CCaMK, and CYCLOPS) and nutrient transporter-encoding genes involved in nutrient exchange at the peri-arbuscular membrane are also regulated by PHR2 (Shi et al. 2021). Overexpression of PHR2 partially restores root colonization by AM fungi in rice and *L. japonicus* under high Pi conditions, where AM symbiosis is typically suppressed in wild-type roots (Das et al. 2022).

Unlike the symbiosis with AM fungi, which occurs under Pi-limiting conditions, Pi deficiency significantly reduces the number of nodules in legumes, hindering nitrogen fixation and overall plant growth and productivity (Hernández et al. 2009, Isidra-Arellano et al. 2020). Significant efforts have been made to understand how legumes cope with Pi deficiency while symbiotically interacting with rhizobia (Hernández et al. 2009, Chen et al. 2011, Suleiman et al. 2013, Cabeza et al. 2014, Nasr Esfahani et al. 2017). Physiological studies conducted on diverse legumes indicate that under Pi deficiency, Pi is preferentially relocated from other plant organs to nodules (Hernández et al. 2009, Cabeza et al. 2014, Nasr Esfahani et al. 2017). Additionally, transcriptional and proteomic studies inform that most of the molecular responses of Pi-deficient root nodules are geared toward efficient Pi use (Hernández et al. 2009, Cabeza et al. 2014, Nasr Esfahani et al. 2017). Furthermore, soybean and *P. vulgaris nark* mutants display no nodule reduction under Pi-deficient conditions, suggesting that the AON system drives reduced nodule numbers under Pi starvation (Isidra-Arellano et al. 2020). AON-related genes contain P1BS *cis*-regulatory elements (Isidra-Arellano et al. 2020), suggesting that PHR1 may participate in controlling the expression of these genes and, hence, play a role in the root nodule symbiosis. PHR positively regulates the Pi homeostasis in soybean nodules under optimal Pi (OPi) conditions (Lu et al. 2020). Thus, we hypothesized that PHR proteins might be a key regulatory component in the root nodule symbiosis, whose genetic roles depend on the plant Pi status.

In the present study, a phylogenetic analysis of PHR and PHL proteins enabled us to classify Phvul006G134700, the same gene reported in our previous study as PvPHR1 (Isidra-Arellano et al. 2020), within the PHL 7 clade. Here, we provide evidence supporting the potential participation of PvPHR-Like7 (PvPHR-L7) in controlling nodule formation in *P. vulgaris*. Modulation of the gene expression of *PvPHR-L7* by RNA interference (RNAi) and overexpression affected nodule formation in *P. vulgaris*. Further whole-genome transcriptional analyses on *PvPHR-L7*-RNAi and *PvPHR-L7*-overexpressing roots with young nodules from control and Pi-deficient *P. vulgaris* plants revealed that PvPHR-L7 may regulate the expression of crucial symbiotic genes involved in nodule development. An expression analysis across different stages of the root nodule symbiosis revealed that the expression of *PvPHR-L7*, *PvNIN*, and *PvTML* is modulated according to the plant host Pi levels. Electromobility shift assays (EMSA) and transactivation assays in *Nicotiana benthamiana* and *P. vulgaris* transgenic roots indicate that PvPHR-L7 modulates the expression of *PvNIN* and *PvTML*. The data presented shed light on the potential role that PvPHR-L7 plays in the root nodule symbiosis.

## Results

### Modulation of the expression of *PvPHR-L7* affects nodule formation in *P. vulgaris*

We previously reported that the gene silencing of Phvul006G134700, which encodes a PHL protein, reduces the expression of *PvNIN*, *PvTML*, *PvRIC1*, and *PvRIC2* in *P. vulgaris* plants grown under Pi-deficient conditions and in the absence of rhizobia (Isidra-Arellano et al. 2020). Further phylogenetic analyses indicate that Phvul006G134700 groups in the clade belonging to PHL 7 from *Arabidopsis thaliana* (Fig. S1). Therefore, we renamed Phvul006G134700 as PvPHR-L7 instead of PvPHR1 as previously reported by Isidra-Arellano et al. (2020). As AtPHR1, PvPHR-L7 contains the MYB and Coiled-Coil domains (Fig. S2C and D), which are required to bind to the P1BS *cis*-regulatory elements and to form dimers, respectively (Rubio et al. 2001, Ried et al. 2021). Moreover, PvPHR-L7 contains the serine residue S216 (Fig. S2C), located in the MYB domain, which is conserved in PHR2, the functional ortholog in rice and *M. truncatula* (Zhou et al. 2008, Wang et al. 2024). The S216 residue is phosphorylated by GS3-SHAGGY-like kinase2 (GSK2) to regulate the PHR activity in *A. thaliana* and rice (Zhang et al. 2024). Likewise, PvPHR-L7 contains the K292, H295, and R302 residues that are required for SPX-Inositol Pyrophosphate8 to bind and control the PHR1 and PHR2 activity under OPi conditions in *A. thaliana* and rice, respectively (Ried et al. 2021) (Fig. S2D). However, PvPHR-L7’s N-terminal is 20 amino acids shorter than the AtPHR1 (Fig. S2B). As PvPHR-L7, MtPHR2 also possesses a 20-amino-acid shorter N-terminal; despite this shorter N-terminal, MtPHR2 is functional and controls the PSRs and the symbiosis with AM fungi in *M. truncatula* (Wang et al. 2021, 2024). AtPHR1 and AtPHR-Like 1 contain a 200-amino-acid longer N-terminal than AtPHR-Like2 and AtPHR-Like3 (Wang et al. 2023). Despite this structural difference in their N-terminal, both AtPHR1/AtPHR-Like1 and AtPHR-Like2/AtPHR-Like3 are fundamental to control the PSRs in *A. thaliana* (Wang et al. 2023). These data indicate that PvPHR-L7 contains crucial domains and motifs required to regulate PHR protein activity in response to the plant’s Pi status. However, we cannot conclude that the absence of 20 amino acids in the PvPHR-L7 N-terminal is not relevant to its activity. Therefore, further investigation is required to determine the potential function of the missing amino acids.

**Figure 1:**
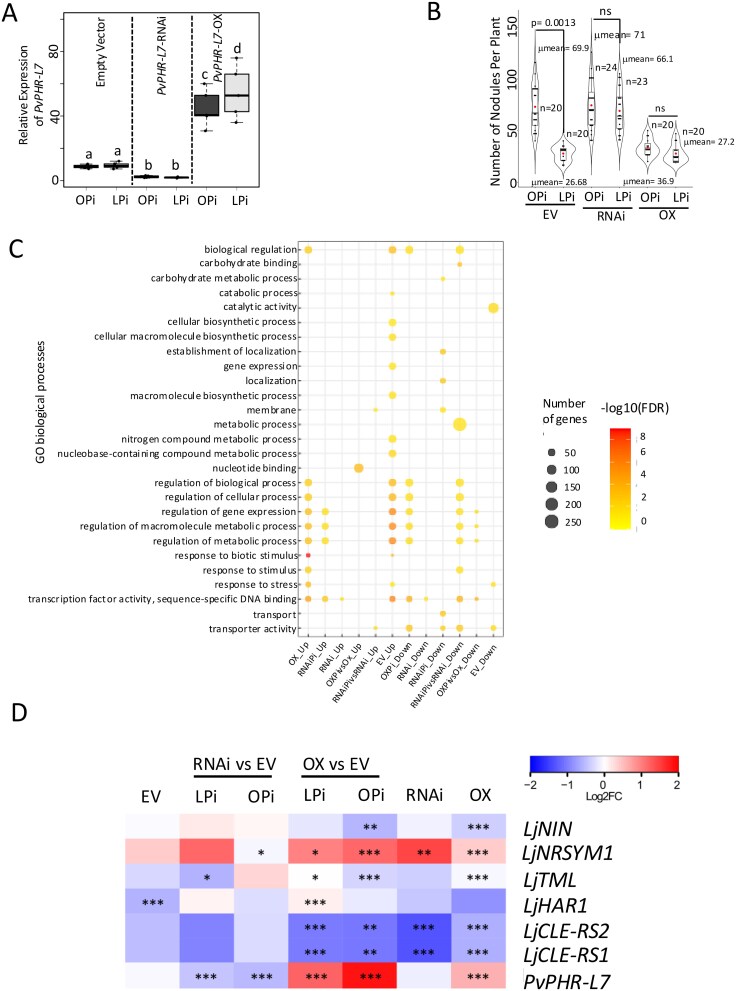
Modulation of the expression of *PvPHR-L7* affects nodule formation and the expression of symbiosis-related genes in *P. vulgaris*. (A) *PvPHR-L7* expression levels in transgenic roots expressing an empty vector or the *PvPHR-L7*-RNAi or *PvPHR-L7*-OX constructs. Box plots represent the first and third quartiles (horizontal box sides) and the minimum and maximum (outside whiskers). Data were obtained from four biological replicates, each containing one transgenic root from 20 independent plants with transgenic roots, resulting in a total of 20 transgenic roots. A one-way analysis of variance (ANOVA) followed by a Tukey honest significant difference (HSD) test was performed (*P*-value < .01). Statistical classes sharing a letter are not significantly different from each other. (B) Nodulation assays in *P. vulgaris* plants with transgenic roots expressing an empty vector (control) or the *PvPHR-L7*-RNAi or *PvPHR-L7*-overexpression (*PvPHR-L7*-OX) constructs. Plants were grown under OPi or LPi conditions. Boxes indicate the second and third quartiles, with the median and mean values represented by lines and red dots, respectively. Nodules were counted only on plants whose root system and nodules showed TdTomato fluorescence. This experiment used at least 20 independent plants from each experimental condition to count mature nodules. Statistical significance was obtained using Welch’s *t*-test. ns: not significant. (C) Enriched GO terms in the differentially regulated genes in: EV_Up: upregulated genes when comparing empty vector OPi vs empty vector LPi. EV_Down: downregulated genes when comparing empty vector OPi vs empty vector LPi. OX_Up: upregulated genes when comparing *PvPHR-L7*-OX LPi vs empty vector LPi. OXPi_Down: downregulated genes when comparing *PvPHR-L7*-OX OPi vs empty vector OPi. RNAi_Up: upregulated genes when comparing *PvPHR-L7*-RNAi LPi vs empty vector LPi. RNAiPi_Up: upregulated genes when comparing *PvPHR-L7*-RNAi OPi vs empty vector OPi. RNAiPi_Down: downregulated genes when comparing *PvPHR-L7*-RNAi OPi vs empty vector OPi. RNAi_Down: downregulated genes when comparing *PvPHR-L7*-RNAi LPi vs empty vector LPi. RNAiPi vs RNAi_Up: upregulated genes when comparing *PvPHR-L7*-RNAi—OPi vs *PvPHR-L7-*RNAi—LPi. RNAiPi vs RNAi_Down: downregulated genes when comparing *PvPHR-L7*-RNAi—OPi vs *PvPHR-L7*-RNAi—LPi. OXPi vs OX_Up: upregulated genes when comparing *PvPHR-L7*-OX—OPi vs *PvPHR-L7*-Ox—LPi. OXPi vs OX_Down: downregulated genes when comparing *PvPHR-L7*-OX—OPi vs *PvPHR-L7*-Ox—LPi. (D) Heatmap showing Log2 fold-change of root nodule symbiosis-related genes (Roy et al. 2020). EV: differentially regulated genes when comparing empty vector—OPi vs empty vector—LPi) conditions. RNAi vs EV: differentially regulated genes when comparing *PvPHR-L7*-RNAi vs empty vector under OPi or LPi conditions. OX vs EV: differentially regulated genes when comparing *PvPHR-L7*-OX vs empty vector under OPi or LPi conditions. RNAi: differentially regulated genes when comparing *PvPHR-L7*-RNAi—OPi vs *PvPHR-L7*-RNAi—LPi (RNAi). OX: differentially regulated genes when comparing *PvPHR-L7*-OX—OPi vs *PvPHR-L7*-OX—LPi (OX). Asterisks indicate different levels of statistical significance of the comparisons (* = adjusted *P*-value < .05; ** = adjusted *P*-value < .01; *** = adjusted *P*-value < .001). Genes with no asterisk are not significantly differentially expressed.

The fact that the gene silencing of *PvPHR-L7* affects the expression of four key root nodule symbiosis-related genes in a non-symbiotic context led us to hypothesize that PvPHR-L7 might be required to modulate the root nodule symbiosis in *P. vulgaris*. To experimentally validate this hypothesis, we assessed the effect of downregulating and overexpressing *PvPHR-L7* in *P. vulgaris* transgenic roots. To this end, we generated plants with transgenic roots expressing an empty vector (control), *PvPHR-L7*-RNAi, or the *PvPHR-L7*-Overexpression (hereafter referred to as *PvPHR-L7*-OX). These *P. vulgaris* plants were grown under optimal- or Pi-deficient conditions. *PvPHR-L7*-RNAi transgenic roots displayed a five-fold reduction in the *PvPHR-L7* transcript levels compared to control plants, whereas *PvPHR-L7*-OX showed a four-fold increase (Fig. 1A).

As anticipated, control plants growing under Pi-deficient conditions developed 60% fewer nodules than plants growing under OPi conditions (Fig. 1B). In contrast, the negative effect of Pi deficiency on nodule formation was not observed in *PvPHR-L7*-RNAi transgenic roots (Fig. 1B). Indeed, *PvPHR-L7*-RNAi plants developed the same number of nodules regardless of the Pi condition (Fig. 1B). In contrast, irrespective of the Pi conditions, *PvPHR-L7*-OX transgenic plants formed the same number of nodules as control plants growing under Pi-deficient conditions (Fig. 1B). In summary, this nodulation assay data indicates that PvPHR-L7 acts as a negative regulator of nodule formation in *P. vulgaris*.

### Overexpression of *PvPHR-L7* reduces the expression of genes required for nodule formation in *P. vulgaris*

White and small nodules (young nodules) are observed after 10 days post-inoculation with rhizobia in *P. vulgaris*, indicating that nodules are immature and unable to fix nitrogen (Fig. S3). Because reduction in the nodule formation is one of the effects of Pi deficiency, we rationalized that analyzing the effects of Pi scarcity in this developmental stage might provide us with transcriptional information of the potential role of *PvPHR-L7* in the nodule formation under Pi-deficient conditions. Hence, we analyzed roots bearing young nodules to capture transcriptional information related to nodule formation. To this end, we performed an RNA-seq analysis in transgenic roots bearing young nodules expressing an empty vector (control), *PvPHR-L7*-RNAi, or *PvPHR-L7*-OX constructs and growing under OPi or deficient Pi conditions. Three biological replicates for each experimental condition were analyzed, resulting in a total of 18 RNA-seq libraries.

Overall, 10.1–12.6 million short sequence reads (2 × 75 bp) were generated from each of 18 RNA-seq libraries, with alignment rates to the reference genome ranging from 89% to 93%. Principal component analysis (PCA) revealed a clustering of biological replicates and variation dependent on silencing or overexpressing, with the first component accounting for 60% of the data variation (Fig. S4A). Comparison of expression levels in each biological condition (relative to the empty vector) revealed 3713 differentially expressed genes (DEGs) with a significant change in expression (adjusted *P* < .05), including 1952 and 1761 genes that were up- or down-regulated, respectively, relative to the empty vector (Table S1). We also observed that *PvPHR-L7*-RNAi and *PvPHR-L7*-OX roots displayed a similar reduction or increase in the *PvPHR-L7* transcript levels as determined by RT-qPCR (Fig. S5). We randomly selected nine genes (Phvul.010G121200, Phvul.001G160100, Phvul.011G080500, Phvul.007G008900, Phvul.004G121666, Phvul.011G163600, Phvul.010G062300, Phvul.007G273200, and Phvul.002G020200) to validate these data sets, and their expression levels were assessed by RT-qPCR. The results show similar trends between RNA-seq and RT-qPCR data as expected (Fig. S4B).

Gene Ontology (GO) enrichment analysis of the DEGs revealed that 29 GO terms were significantly enriched (Fig. 1C). Among the enriched GO terms were transcription factor activity, regulation of gene expression, transport activity, and response to biotic stimulus (Fig. 1C). Interestingly, the transcription factor activity and gene expression were the GO terms that contained more DEG than other terms (Fig. 1C). Thus, this analysis informs the relevance of PvPHR-L7 as a transcriptional regulator in the root nodule symbiosis in *P. vulgaris*.

To gain insight into the potential participation of PvPHR-L7 in the nodule formation, we compared the transcriptomes of *PvPHR-L7-*RNAi and *PvPHR-L7*-OX roots against the empty vector roots’ transcriptome. The transcriptional data sets from this analysis were used to identify whether the expression of any of the 200 symbiotic genes reported by Roy et al. (2020) was affected by the gene silencing or overexpression of *PvPHR-L7*. This analysis revealed that the downregulation and overexpression of *PvPHR-L7* affected the expression of various genes involved in diverse stages of the root nodule symbiosis, including rhizobial infection, nodule organogenesis, nodule metabolism and transport, and AON (Fig. S6 and Table S2). For instance, the expression of *TML*, *CLE-RS1*, and *CLE-RS2*, three crucial components of the AON pathway, was significantly downregulated in *PvPHR-L7*-OX roots (Fig. 1D). We also observed the downregulation of *TML* in Pi-deficient *PvPHR-L7*-RNAi roots (Fig. 1D), which is in line with the presence of the P1BS *cis*-regulatory element in its promoter region (Isidra-Arellano et al. 2020). However, we did not expect this downregulation in *PvPHR-L7*-OX roots, and we hypothesize that this effect may be due to the ectopic expression of *PvPHR-L7*. Moreover, the expression of *NIN*, which coordinates nearly every step of the root nodule symbiosis (Schauster et al. 1999, Marsh et al. 2007, Liu et al. 2019, Feng et al. 2021), was significantly downregulated in *PvPHR-L7*-OX roots, whereas *PHR-L7*-RNAi roots displayed a trend to be induced although not statistically significant (Fig. 1D). Moreover, we observed that the overexpression of *PvPHR-L7* decreased the expression of *cytokinin receptor 1* (*CRE1*), *interacting protein of DMI3* (*IPT3*), *cytokinin oxidase/dehydrogenase3* (*CKX3*), *basic helix–loop–helix 1* (*BHLH1*), *auxin response factor 8a* (*ARF8a*), *ARF8b*, and *scarecrow-like13 involved in nodulation 1* (*SIN1*), which all are essential for nodule development (Fig. S6; Tirichine et al. 2007, Godiard et al. 2011, Battaglia et al. 2014, Sasaki et al. 2014, Wang et al. 2015, Reid et al. 2016). Similarly, regardless of the Pi conditions, the expression of genes encoding diverse types of sugar and mineral nutrients transporters was downregulated in roots bearing young nodules expressing *PvPHR-L7*-RNAi or the *PvPHR-L7*-OX construct (Fig. S6 and Table S2). Conversely, the expression of genes inhibiting nodulation (i.e. *Nitrate Unresponsive Symbiosis 1*: *NRSYM1*; *Calcium Dependent Proteinase 3*: *CPK3*, and *Shaggy Like Kinase 1*: *LSK1*) and leading to symbiotic host restriction (i.e. *Rj4*) was upregulated in *PvPHR-L7*-OX roots bearing young nodules (Fig. 1D and Fig. S6). Further comparison between *PvPHR-L7*-RNAi and *PvPHR-L7*-OX transgenic roots bearing young nodules confirmed that the expression of most symbiotic genes promoting nodulation is reduced when *PvPHR-L7* is overexpressed (Fig. 1D and Fig. S6). As we mentioned earlier, the significant effect on the expression of symbiotic genes when *PvPHR-L7* is overexpressed may be due to an effect of the ectopic expression of *PvPHR-L7*. Altogether, our transcriptome data suggest that PvPHR-L7 regulates the expression of symbiotic genes required to form a root nodule, including *NIN*, *TML*, *CLE-RS1*, and *CLE-RS2*. Our transcriptomic dataset provides evidence supporting the participation of PvPHR-L7 in the nodule formation in *P. vulgaris*.

### Low Pi affects the expression of symbiotic genes

**Figure 2:**
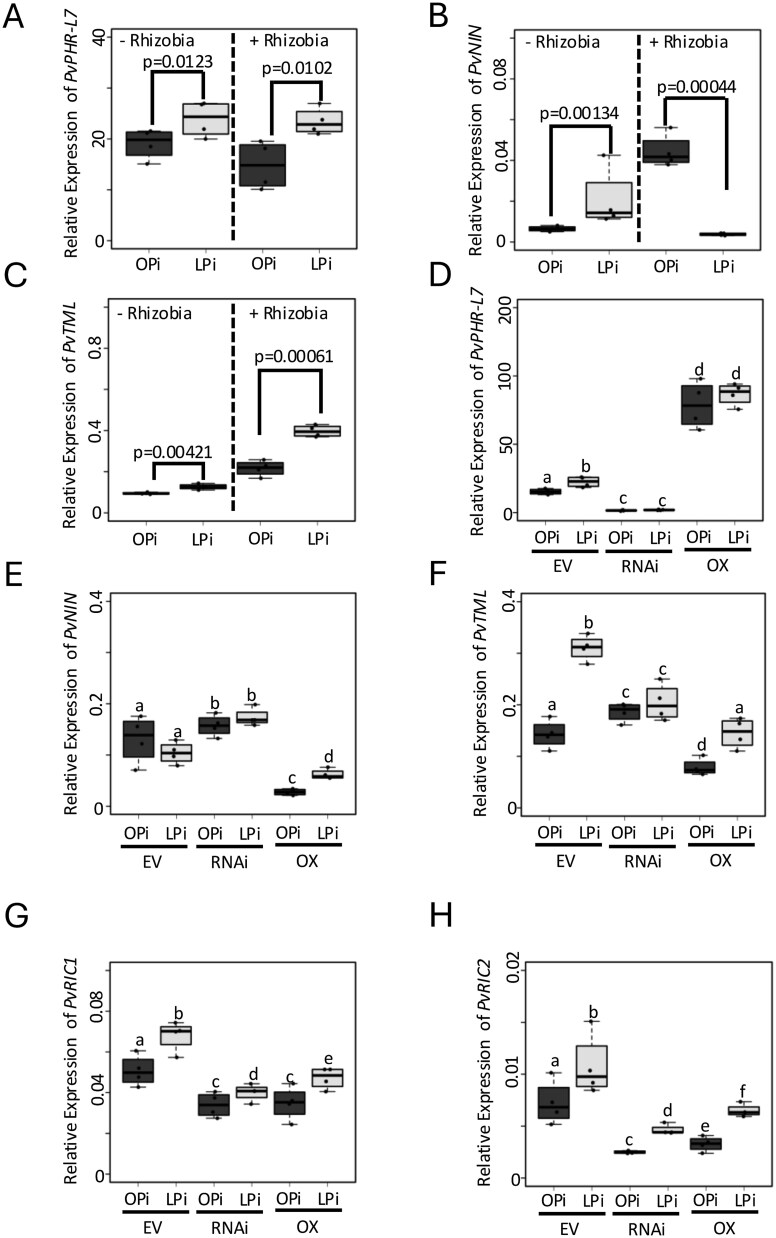
Pi deficiency through PvPHR-L7 affects the expression of symbiotic genes. The expression of *PvPHR-L7* (A), *PvNIN* (B), and *PvTML* (C) was evaluated in roots growing under OPi or LPi conditions and in the absence or presence of rhizobia. Data were obtained from four biological replicates. Statistical significance was obtained using Welch’s *t*-test. To evaluate the potential participation of PvPHR-L7 in controlling the expression of symbiotic genes, the expression of *PvNIN* (E), *PvTML* (F), *PvRIC1* (G), and *PvRIC2* (H) was evaluated in *P. vulgaris* transgenic roots bearing young nodules and expressing and empty vector (EV, control), or the *PvPHR-L7*-RNAi (RNAi) or *PvPHR-L7*-OX (OX) constructs. Silencing and overexpression levels of *PvPHR-L7* were also evaluated (D). Data were obtained from four biological replicates. A one-way ANOVA followed by a Tukey HSD test was performed (*P*-value < .01). Statistical classes sharing a letter are not significantly different from each other. Box plots represent the first and third quartiles (horizontal box sides) and the minimum and maximum (outside whiskers).

Pi deficiency increases the expression of *PvNIN*, *PvRIC1*, *PvRIC2*, and *PvTML*, which are essential components of the AON pathway, under non-symbiotic conditions with rhizobia in *P. vulgaris* (Isidra-Arellano et al. 2020). In this study, we observed that Pi scarcity also affects the expression of these symbiotic genes in *PvPHR-L7*-RNAi and *PvPHR-L7*-OX roots bearing young nodules, suggesting that the host plant’s Pi status may be fundamental in activating the expression of symbiotic genes, likely through the action of PvPHR-L7. To experimentally test this hypothesis, we assessed the expression of *PvNIN*, *PvTML*, and *PvPHR-L7* by RT-qPCR under both non-symbiotic and symbiotic conditions with rhizobia and plants grown under OPi- or low-Pi (LPi) conditions. As expected, LPi increases the expression of *PvPHR-L7*, *PvNIN,* and *PvTML* in a non-symbiotic scenario (Fig. 2A–C). However, we observed that Pi deficiency significantly reduces the expression of *PvNIN* when plants symbiotically interact with rhizobia (Fig. 2B). In contrast, the expression of *PvPHR-L7* and *PvTML* increases under LPi conditions while interacting with rhizobia (Fig. 2A and C). Although Pi deficiency alone increases the expression of *PvNIN* and *PvTML*, their expression levels are lower than those observed in rhizobia-inoculated roots growing under OPi conditions (Fig. 2A–C). Next, we evaluated whether the reduction in the expression of *PvNIN* or the upregulation of *PvTML* and *PvPHR-L7* occurred across the root nodule symbiosis. We observed that the expression of *PvNIN* was significantly reduced during nodule development (Fig. S7). In contrast, the expression of *PvTML* and *PvPHR-L7* was upregulated in the three tested nodule developmental stages (Fig. S7). These transcriptional analyses confirm that Pi scarcity increases the expression of *PvNIN* and *PvTML* in the absence of rhizobia. Moreover, this transcriptional data also provides evidence that host plant Pi status is fundamental in activating the transcription of *PvNIN*, the master regulator of the root nodule symbiosis.

Our RNA-seq analysis revealed that gene silencing and overexpression of *PvPHR-L7* affect the expression of *NIN*, *TML*, *CLE-RS1*, and *CLE-RS2* (Fig. 1D). These transcriptional data suggest that PvPHR-L7 may be a key regulator of these symbiotic genes. To further investigate this hypothesis, we validated their expression by RT-qPCR in control, *PvPHR-L7*-RNAi, and *PvPHR-L7*-OX roots bearing young nodules and grown under OPi or deficient Pi conditions. We evaluated the expression of *PvRIC1* and *PvRIC2*, which are the orthologous genes of *CLE-RS1* and *CLE-RS2*, respectively (Isidra-Arellano et al. 2020). As expected, *PvPHR-L7*-RNAi transgenic roots displayed a 10-fold reduction in the *PvPHR-L7* transcript levels compared to control plants, whereas *PvPHR-L7*-OX showed a five-fold increase, confirming the gene silencing and overexpression of *PvPHR-L7*, respectively (Fig. 2D). Likewise, we observed that the expression pattern of these four genes was like those obtained by the RNA-seq analysis (Figs 1D and 2D–H). We also observed that regardless of the Pi status, *PvPHR-L7*-RNAi roots displayed an increase in the expression of *PvNIN*, whereas *PvPHR-L7*-OX roots showed a four-fold reduction in the expression of this gene (Fig. 2E). In contrast, we observed a significant decrease in the expression levels of *PvTML*, *PvRIC1*, and *PvRIC2* in both *PvPHR-L7*-RNAi and *PvPHR-L7*-OX roots irrespective of the Pi condition compared to empty vector roots (Fig. 2F–H). The reduction in the expression of *PvTML*, *PvRIC1*, and *PvRIC2* in *PvPHR-L7*-OX roots was unexpected, as these genes possess P1BS *cis*-regulatory elements in their promoter regions. The expression pattern of these AON-related genes in *PvPHR-L7*-OX roots suggests that the expression of *PvPHR-L7* must be tightly regulated to avoid adverse effects on the transcriptional activity of potential PvPHR-L7 target genes. Despite this unexpected expression pattern in *PvPHR-L7*-OX roots, we observed a significant induction in the expression of these four symbiotic genes in response to Pi deficiency in *PvPHR-L7*-OX roots; however, their expression levels were reduced compared to empty vector plants (Fig. 2F–H). Altogether, this transcriptional analysis confirms that OPi levels are required to appropriately respond to rhizobia and activate the expression of genes involved in the root nodule symbiosis. Moreover, these data also add experimental evidence supporting the participation of *PvPHR-L7* as a genetic regulator of the root nodule symbiosis, most likely as a negative regulator.

### PvPHR-L7 can transactivate the expression of *PvNIN* and *PvTML* in *P. vulgaris* roots in the absence of rhizobia

AtPHR1, along with PHL proteins, binds to the P1BS *cis*-regulatory element present in the promoter region of numerous Pi deficiency-responsive genes (Bustos et al. 2010, Wang et al. 2023). Moreover, PHR2 is also required to activate the expression of genes necessary for establishing symbiosis with AM fungi in rice and *L. japonicus* (Rubio et al. 2001, Shi et al. 2021, Das et al. 2022). *PvTML1* and *PvNIN* contain at least one P1BS *cis*-regulatory element in their promoter regions (Fig. 5A). The presence of the P1BS *cis*-regulatory element and the fact that the LPi increases the expression of *PvNIN* and *PvTML* in the absence of rhizobia and that the gene silencing and overexpression of *PvPHR-L7* affect the expression of these symbiotic genes led us to hypothesize that the overexpression of *PvPHR-L7* might transactivate the expression of *PvTML* and *PvNIN* in a non-symbiotic context. To validate this hypothesis, we first undertook transactivation assays in *N. benthamiana* leaves (Fig. S8). The transcriptional fusions *pPvTML:GUS* or *pPvNIN:GUS* were co-infiltrated with 3*5S:PvPHR-L7* into *N. benthamiana* leaves via agroinfiltration. We used RT-qPCR to evaluate *GUS* transcript levels and the intensity of GUS activity, thereby monitoring the transcriptional activity of the *PvTML* and *PvNIN* promoters. Our RT-qPCR analyses confirmed that infiltrated leaves with 3*5S:PvPHR-L7* alone or in combination with the transcriptional fusions displayed overexpression of *PvPHR-L7* (Fig. S8A). We observed that cells expressing *pPvTML:GUS* or *pPvNIN:GUS* alone showed no *GUS* expression or activity (Fig. S8A and B). In contrast, cells co-expressing *35S:PvPHR-L7* and *pPvTML:GUS* or *pPvNIN:GUS* displayed *GUS* expression and activity (Fig. S8A and B). These transactivation assays in *N. benthamiana* indicate that PHR-L7 can transactivate the expression of *PvNIN* and *PvTML* in a non-symbiotic scenario.

**Figure 3:**
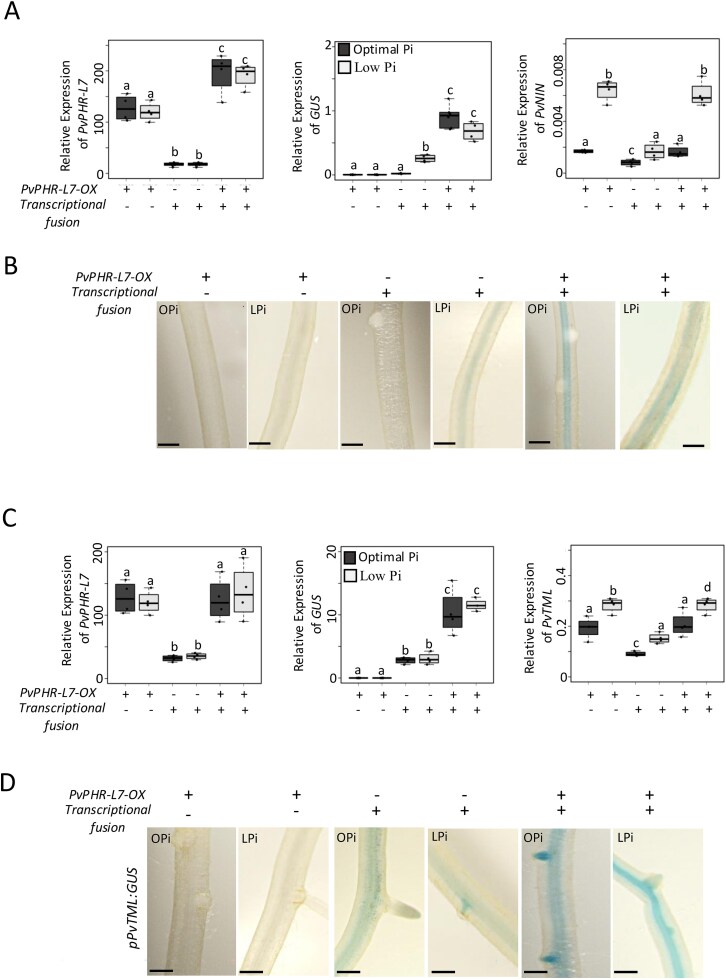
Overexpression of *PvPHR-L7* activates the expression of *PvNIN* and *PvTML* in non-symbiotic conditions with rhizobia. Transactivation assays in *P. vulgaris* transgenic roots under non-symbiotic conditions with rhizobia were assessed by RT-qPCR (A and C) or by GUS activity (B and D). Expression levels of endogenous *PvNIN* and *PvTML* were also evaluated by RT-qPCR (A and C). *pPvNIN:GUS* and *pPvTML:GUS* constructs alone or in combination with a *35S:PvPHR-L7-MYC* (*PvPHR-L7-OX*) construct were co-infected in *P. vulgaris* transgenic roots. Transgenic roots were analyzed after 15 days post-infection with *A. rhizogenes*. Roots were incubated in GUS histochemical staining buffer at 37°C for 4–6 h for *pPvNIN:GUS* activity, whereas 30 min for *pPvTML:GUS*. Scale bars in panels B and D represent 1 mm. Images represent 10 biological replicates, each containing 10 transgenic roots. Box plots in panels A and C represent the first and third quartile (horizontal box sides) and the minimum and maximum (outside whiskers). Data were obtained from four biological replicates. A one-way ANOVA followed by a Tukey HSD test was performed (*P*-value < .01). Statistical classes sharing a letter are not significantly different from each other.

Next, we generated *P. vulgaris* transgenic roots expressing the *pPvTML:GUS* or *pPvNIN:GUS* constructs alone or in combination with *35S:PvPHR-L7* and growing under OPi conditions and in the absence of rhizobia. We used RT-qPCR to assess the *GUS* and endogenous *PvNIN* and *PvTML* transcript levels. We also evaluated GUS’s activity to confirm our transcriptional observations. Our RT-qPCR analyses confirmed that roots expressing 3*5S:PvPHR-L7* alone or in combination with the transcriptional fusions displayed overexpression of *PvPHR-L7*; however, we did not observe an increase in the expression levels of *PvPHR-L7* in response to Pi deficiency. The lack of upregulation of PvPHR-L7 in Pi-deficient roots might be due to the strong effect of the *35S* promoter used in this study. (Fig. 3A and C). We observed that *pPvNIN:GUS* roots growing under OPi conditions displayed no GUS expression, whereas roots expressing *35S:PvPHR-L7* and *pPvNIN:GUS* showed a strong GUS expression (Fig. 3A). In contrast, roots expressing *pPvTML:GUS* alone displayed a faint *GUS* expression and activity, which was significantly enhanced when *PvPHR-L7* was co-expressed (Fig. 3C). We also evaluated the GUS activity in roots expressing the transcriptional fusions alone or in combination with *35S:PvPHR-L7* to confirm the expression levels of *GUS* observed in each experimental condition. We observed that *35S:PvPHR-L7* roots and those expressing *pPvNIN:GUS* alone displayed no GUS activity, whereas transgenic roots co-expressing *35S:PvPHR-L7* and *pPvNIN:GUS* showed GUS staining (Fig. 3B). Unlike *pPvNIN:GUS* roots, transgenic roots expressing the *pPvTML:GUS* construct alone displayed GUS activity, which was significantly enhanced when *PvPHR-L7* was overexpressed (Fig. 3D). These GUS activity assays confirmed what we observed by RT-qPCR. Finally, we evaluated the endogenous *PvNIN* and *PvTML* expression levels by RT-qPCR. This transcriptional analysis revealed that, regardless of the presence of the transcriptional fusions, the overexpression of *PvPHR-L7* significantly increased the expression of the endogenous *PvNIN* and *PvTML* (Fig. 3A and C), indicating that the overexpression of *PvPHR-L7* transactivates both transcriptional fusions and endogenous genes.

To evaluate the effect of Pi scarcity on the expression of *PvNIN* and *PvTML*, we performed a similar transactivation assay but under LPi conditions. We observed that roots expressing *pPvNIN:GUS* alone displayed a faint but consistent GUS expression and activity, which was significantly enhanced when *PvPHR-L7* was co-expressed (Fig. 3A and B). A similar expression pattern was observed in roots expressing *PvTML:GUS* alone or co-expressing *35S:PvPHR-L7* and *pPvTML:GUS* (Fig. 3C and D). To further validate our observation, we evaluated the effect of overexpressing *PvPHR-L7* on the transcriptional activity of the endogenous *PvNIN* and *PvTML* by RT-qPCR (Fig. 3A and C). This transcriptional analysis revealed that, regardless of the presence of the transcriptional fusions, the overexpression of *PvPHR-L7* upregulates the expression of endogenous *PvNIN* and *PvTML*. Indeed, the expression levels of these endogenous genes were higher in Pi-deficient roots than those observed in roots from plants growing under OPi conditions (Fig. 3A and C).

Although our transactivation assays indicate that the overexpression of *PvPHR-L7* activates the transcriptional fusions and endogenous genes, we observed differences between the *GUS* and the endogenous gene expression levels. For instance, regardless of the Pi status, the overexpression of *PvPHR-L7* transactivated at the same level the expression of *pPvNIN:GUS* and *pPvTML:GUS* (Fig. 3A and C). In contrast, the overexpression of *PvPHR-L7* had a greater impact on the expression of the endogenous *PvNIN* and *PvTML* under Pi-deficient conditions than in OPi conditions. Despite this difference, the expression levels of endogenous *PvNIN* and *PvTML* under OPi conditions were greater than those observed in roots expressing the transcriptional fusions alone (Fig. 3A and C). These differences in the effect of the overexpression of *PvPHR-L7* on the transcriptional activity of the endogenous *PvNIN* and *PvTML* under OPi conditions might be due to the presence of additional *cis*-regulatory elements in the native promoters that were not located in the promoter regions used in this study. Altogether, these transcriptional analyses add additional experimental evidence to support the hypothesis that PvPHR-L7 promotes the expression of these symbiotic genes in a non-symbiotic scenario.

### Overexpression of *PvPHR-L7* negatively regulates the expression of *PvNIN* and *PvTML* under symbiotic conditions with rhizobia

**Figure 4:**
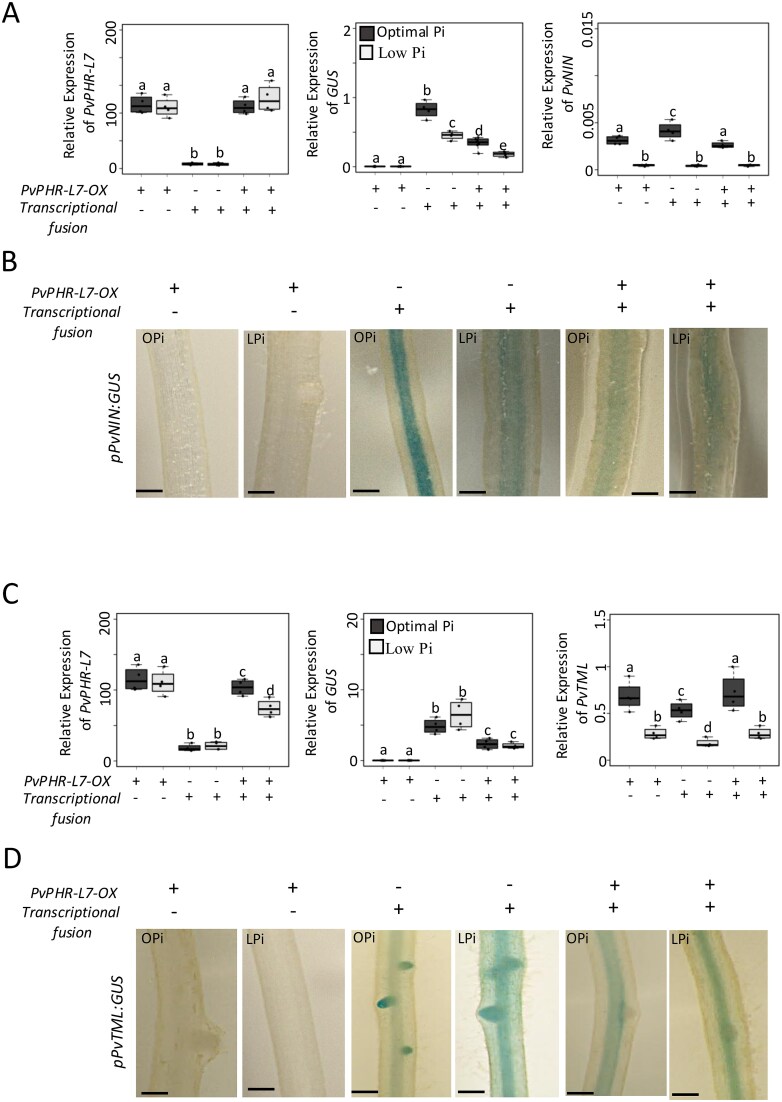
Overexpression of *PvPHR-L7* reduces the expression of *PvNIN* and *PvTML* in symbiotic conditions with rhizobia. Transactivation assays of *P. vulgaris* transgenic roots under symbiotic conditions with rhizobia were evaluated by RT-qPCR (A and C) or by GUS activity (B and D). Expression levels of endogenous *PvNIN* and *PvTML* were also assessed by RT-qPCR (A and C). *pPvNIN:GUS* and *pPvTML:GUS* constructs alone or combined with a *35S:PvPHR-L7-MYC* construct were co-infected in *P. vulgaris* transgenic roots. Transgenic roots in symbiosis with rhizobia were assayed for GUS expression and activity 6 days post-rhizobia inoculation. Roots were incubated in GUS histochemical staining buffer at 37°C for 4–6 h for *pPvNIN:GUS* activity, whereas 30 min for *pPvTML:GUS*. Scale bars represent 1 mm. Images represent 10 biological replicates, each containing 10 transgenic roots. Box plots in panel A represent the first and third quartiles (horizontal box sides) and the minimum and maximum (outside whiskers). Data were obtained from four biological replicates. A one-way ANOVA followed by a Tukey HSD test was performed (*P*-value < .01). Statistical classes sharing a letter are not significantly different from each other.

Our transcriptional data indicate that LPi reduces the expression of *PvNIN* while interacting with rhizobia (Fig. 2). In contrast, the expression of *PvTML* is increased in the same nutritional conditions (Fig. 2). Our transactivation assays and transcriptional data indicate that PvPHR-L7 activates the expression of these root nodule symbiosis-related genes, and that this increase is greater under Pi-deficient conditions and without rhizobia (Fig. 3A and C). These data led us to hypothesize that the transcriptional activity of *PvPHR-L7* must be tightly regulated when *P. vulgaris* interacts with rhizobia. To test this hypothesis experimentally, we undertook transactivation assays in *P. vulgaris* roots under Opi and LPi conditions and interacting with rhizobia. Our RT-qPCR analyses confirmed that roots expressing 3*5S:PvPHR-L7* alone or combined with the transcriptional fusions displayed overexpression of *PvPHR-L7* (Fig. 4A and C). As by non-symbiotic conditions with rhizobia, we did not observe an increase in the expression of PvPHR-L7 in Pi-deficient roots, which could be due to the strong effect of the *35S* promoter used in this study. Six-days rhizobia-inoculated roots expressing *pPvNIN:GUS* or *pPvTML*:GUS alone and growing under OPi displayed *GUS* expression and activity, which was reduced or increased under LPi conditions (Fig. 4A and B), respectively, confirming what we observed in rhizobia-inoculated roots and different stages of the nodule development (Fig. 2 and Fig. S7). Interestingly, when *35S:PvPHR-L7* was co-expressed along with *pPvNIN:GUS* or *pPvTML*:GUS, we observed, regardless of the plant host’s Pi status, a significant reduction in the *GUS’s* transcriptional activity (Fig. 4A–C). This reduction in the *GUS*’s transcript levels was further evaluated by the activity of this reporter protein. As expected, we observed a decrease in the GUS activity in *pPvNIN:GUS* roots growing under LPi conditions and inoculated with rhizobia (Fig. 4B). Likewise, we observed an increase in the GUS activity in Pi-deficient *pPvTML:GUS* roots (Fig. 4D). However, when *PvPHR-L7* was co-expressed, regardless of the Pi status, we observed a reduced GUS activity in *pPvNIN:GUS* and *pPvTML:GUS* roots. Thus, these GUS assays confirm what we observed at the transcriptional level.

To confirm the effect of overexpressing *PvPHR-L7* on transcriptional activity of the promoters of *PvNIN* and *PvTML*, we evaluated the expression levels of the endogenous *PvNIN* and *PvTML* in transgenic roots co-expressing *35S:PvPHR-L7* and *pPvNIN:GUS* or *pPvTML:GUS* (Fig. 4A and C). Although we observed a similar transcriptional activity between *pPvNIN:GUS* and the endogenous *PvNIN*, we detected differences between them (Fig. 4C). We observed that, unlike *pPvTML:GUS*, the transcription of the endogenous *PvTML* decreases in LPi conditions regardless of the overexpression of *PvPHR-L7* (Fig. 4C). Moreover, we observed that the overexpression of *PvPHR-L7* slightly, but statistically significantly, increases the expression of the endogenous *PvTML* in both OPi and LPi conditions (Fig. 4C). Despite these differences, we observed a significant reduction in the transcriptional activity of *PvTML* under Pi-deficient conditions while symbiotically interacting with rhizobia. Altogether, these data indicate that the host plant’s Pi status is fundamental in controlling the expression of *PvNIN* and *PvTML* while interacting with rhizobia. These data also suggest that the spatiotemporal activity of PvPHR-L7 must be tightly regulated to control the expression of symbiotic genes according to the host plant’s Pi status.

### PvPHR-L7 can bind to the promoter region of *PvNIN* and *PvTML*

**Figure 5:**
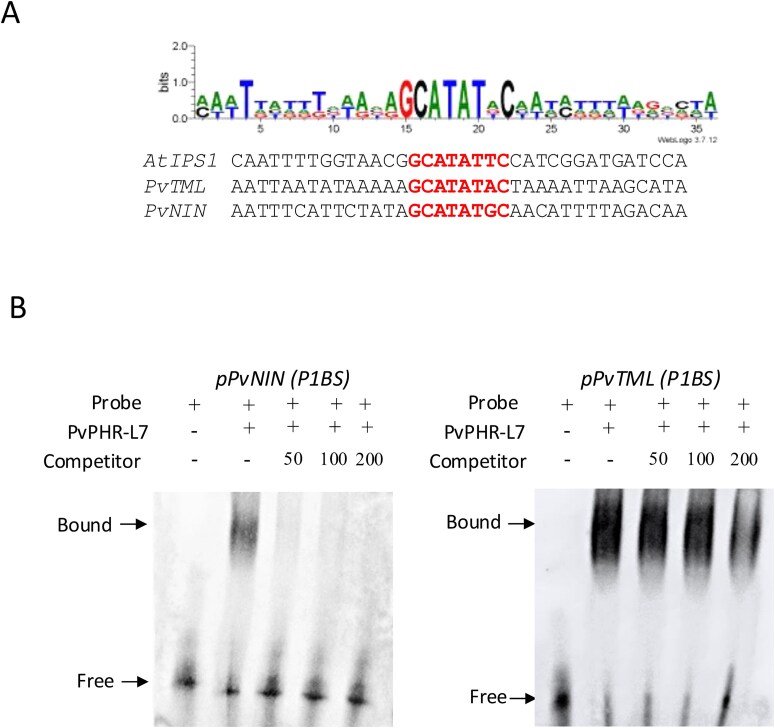
The recombinant PvPHR-L7 protein binds to the promoters of *PvNIN* and *PvTML*, which contain a P1BS *cis*-regulatory element. (A) *PvTML* and *PvNIN* contain P1BS *cis*-element in their promoter regions. (B) PvPHR-L7 binds to the promoter regions of *PvNIN* and *PvTML*. Biotin-labeled probes of *PvNIN* or *PvTML* were incubated with recombinant PvPHR-L7. Competition analyses were performed by adding different concentrations (50, 100, or 200 nM) of unlabeled oligo-DNAs. Images are representative of four biological replicates.

The fact that PvPHR-L7 transactivates the expression of *PvNIN* and *PvTML* in a non-symbiotic scenario and that their promoter regions contain at least one P1BS *cis*-regulatory element led us to evaluate the capability of PvPHR-L7 to bind to the promoter regions of these genes. To validate this hypothesis experimentally, we undertook Electrophoresis Mobility Shift Assays (EMSA) by using a recombinant PvPHR-L7 protein and promoter regions from *PvTML* and *PvNIN* containing the P1BS *cis*-regulatory element (Fig. 5A). The recombinant PvPHR-L7 protein binds to P1BS *cis*-element sites in the *PvTML* and *PvNIN* promoter regions (Fig. 5B). Adding unlabeled competitor probes outcompeted the binding of PvPHR-L7 to their promoter, confirming the specificity of PvPHR-L7 binding (Fig. 5B). This *in vitro* assay suggests that PvPHR-L7 might participate in controlling the expression of *PvNIN* and *PvTML*. However, *in planta* assays, such as chromatin immunoprecipitation (ChIP)-qPCR, remain to be performed in the endogenous Phaseolus root context.

## Discussion

Pi deficiency significantly reduces nodule formation and nitrogen-fixation activity in diverse legumes (Hernández et al. 2009, Chen et al. 2011, Suleiman et al. 2013, Cabeza et al. 2014, Nasr Esfahani et al. 2017). Our previous data indicate that Pi scarcity reduces the number of nodules through the activation of the AON pathway in *P. vulgaris* and *Glycine max*, hence making the NARK receptor a pivotal component to regulate nodule formation under Pi-deficient conditions in these legumes (Isidra-Arellano et al. 2020). Furthermore, gene silencing of *PvPHR-L7* reduces the expression of *PvNIN*, *PvRIC1/2*, and *PvTML* under non-symbiotic conditions with rhizobia (Isidra-Arellano et al. 2020). This previous data suggests the potential participation of PvPHR-L7 as a regulator of nodule formation in *P. vulgaris*. In the present study, we provide different lines of evidence suggesting an intricate interplay between PvPHR-L7 and the expression of symbiotic genes, shedding light on its role in root nodule symbiosis in *P. vulgaris*.

*NIN* and *TML* transcriptional activity is tightly modulated to ensure a successful symbiosis with rhizobia. Failure of their transcriptional activity can lead to defects in nodule development, from no nodule formation to a hyper-nodulation phenotype (Takahara et al. 2013, Soyano et al. 2014). For instance, optimal nitrate levels promote the interaction between NIN-Like Protein 4 (NLP4) or NLP1 and NIN, preventing NIN from activating the expression of its target genes, thereby inhibiting nodule formation (Nishida et al. 2018, 2021). Here, we provide evidence that under symbiotic conditions with rhizobia, Pi deficiency reduces the expression of *PvNIN* while enhancing the expression of *PvTML*, the negative regulator of nodule formation (Takahara et al. 2013). Hence, our transcriptional data revealed that the plant host Pi level is fundamental in regulating the transcriptional activity of crucial symbiotic genes for the root nodule symbiosis in *P. vulgaris*.

Pi deficiency increases the expression of *PvNIN* and *PvTML* in the absence of rhizobia (Isidra-Arellano et al. 2020). In this study, we further confirmed through transactivation assays that Pi deficiency in the absence of rhizobia increases the expression of *PvNIN* and *PvTML* via the action of PvPHR-L7. The potential direct binding of PvPHR-L7 in regulating the expression of *PvNIN* and *PvTML* was partially validated by *in vitro* EMSA assays, which must be further validated by *in planta* assays (e.g. ChIP-qPCR). Our data suggest that *PvNIN* and *PvTML* may play a role in *P. vulgaris* responses to Pi deficiency in a non-symbiotic scenario, likely by controlling the expression of genes that participate in modulating root architecture. This hypothesis is supported by the fact that NIN controls lateral root development in plants belonging to the Nitrogen Fixation Clade (NFC) (Schiessl et al. 2019, Soyano et al. 2019, Dong et al. 2020). Indeed, the lateral root development genetic circuit was rewired to support nodule development (Schiessl et al. 2019, Soyano et al. 2019, Dong et al. 2020). Moreover, NIN is retained in plant species outside the NFC, such as Populus sp., where a homologous NIN controls lateral root development (Irving et al. 2022). These previously reported data support the hypothesis that NIN might have an ancestral role in root development. Despite this evidence, further investigation is required to understand the potential non-symbiotic role of NIN and TML in Pi deficiency conditions.

In the present study, we extended our transcriptional analysis of these symbiotic genes in response to Pi deficiency while interacting with rhizobia. We also observed that Pi deficiency increases the expression of *PvTML*, a negative regulator of the root nodule symbiosis, while reducing the expression of the master regulator of this symbiosis, *PvNIN*. Our transactivation assays under symbiotic conditions with rhizobia revealed that the overexpression of *PvPHR-L7* represses the expression of *PvNIN* and, to lesser extent of *PvTML*. This transactivation assay data aligns with our RNA-seq data, which indicates that under symbiosis with rhizobia, the overexpression of *PvPHR-L7* decreases the expression of *PvNIN* and *PvTML*. It was unexpected to observe a decrease in the expression of *PvTML* in *PvPHR-L7*-OX roots, given the presence of P1BS *cis*-regulatory elements in its promoter region and the fact that this gene is a negative regulator of nodule formation. This unexpected expression can be due to the ectopic expression of *PvPHR-L7*. Moreover, this expression pattern provides additional evidence supporting that the activity of PvPHR-L7 must be tightly spatiotemporally regulated to avoid adverse effects on nodule development. Altogether, these data provide additional evidence supporting the hypothesis that Pi deficiency promotes the expression, likely through PvPHR-L7, of *PvTML* while decreasing the expression of *PvNIN*, thereby restricting the root nodule symbiosis under Pi scarcity conditions. Moreover, these data provide experimental evidence supporting the involvement of the AON pathway in regulating root nodule symbiosis under Pi-deficient conditions, as we previously suggested (Isidra-Arellano et al. 2020).

In this study, we observed that *PvPHR-L7*-RNAi roots bearing young nodules displayed a reduction in the expression of *PvTML*. However, we did not observe a decrease in the expression of *PvNIN;* instead, the gene silencing of *PvPHR-L7* increased its expression. This difference in the transcriptional activity of *PvNIN* in non-symbiotic and symbiotic conditions with rhizobia led us to hypothesize that under symbiotic conditions with rhizobia, PvPHR-L7 may act as a repressor of *PvNIN*. This hypothesis is partially supported by the fact that *PvPHR-L7*-OX roots bearing young nodules display a reduction in the expression of *PvNIN*. However, further experimentation is required to determine whether, under symbiotic conditions with rhizobia, PvPHR-L7 directly represses the expression of *PvNIN* or indirectly through the activation of yet-unknown repressors. Furthermore, the expression pattern of *PvNIN* and *PvTML* might partially explain the lack of reduction of the number of nodules in *PvPHR-L7*-RNAi regardless of the Pi status. Unexpectedly, the overexpression of *PvPHR-L7* reduced the expression of AON-related genes and *PvNIN*. This unexpected expression of AON-related genes suggests that the transcriptional activity of *PvPHR-L7* must be tightly spatiotemporal regulated to control the nodule formation according to the host plant’s Pi status.

Pi deficiency promotes the symbiosis between land plants and AM fungi, where PHR2 is a central regulator activating the expression of diverse genes participating in strigolactone biosynthesis, perception, and decoding AM fungi-derived LCOs in both legume and non-legume (Shi et al. 2021, Das et al. 2022). In contrast, LPi reduces the number of nodules in diverse legumes (Hernández et al. 2009, Chen et al. 2011, Suleiman et al. 2013, Cabeza et al. 2014, Nasr Esfahani et al. 2017). In this study, we provided evidence indicating that this negative effect is not observed in *PvPHR-L7*-RNAi transgenic roots. In contrast, the overexpression of this transcription factor reduces the number of nodules regardless of the Pi conditions. In line with this phenotype, our RNA-seq data revealed that the overexpression of *PvPHR-L7* reduces the expression of crucial genes required to form a nodule. This data adds additional evidence supporting that PvPHR-L7 might act as a negative regulator of nodule formation by regulating the expression of critical genes involved in nodule development in *P. vulgaris*. Furthermore, these data also add more evidence supporting a model where PvPHR-L7 integrates the plant Pi host levels to control the expression of essential symbiotic genes required for nodule formation and avoid an energetic imbalance.

## Materials and Methods

### Plant material

*P. vulgaris* wild-type cultivar Negro Jamapa was used in this study. Seeds were sterilized using the method described in Isidra-Arellano et al. (2018) and subsequently germinated for 2 days at a temperature of 25°C in Petri dishes containing sterile germination paper (Anchor PCO, St. Paul, MN, USA) in the dark.

### Bacterial strain and culture conditions

The *Rhizobium tropici* CIAT 899 strain was used to inoculate *P. vulgaris* wild-type and plants with transgenic roots. The *R. tropici* cells were grown on P.Y. medium (5 g/l peptone and 3 g/l yeast extract), supplemented with 0.7 M CaCl_2_ and 20 μg/ml nalidixic acid, at 30°C for 2 days. The cells were then harvested and suspended in sterile water to a concentration of OD_600_ = 0.3. One milliliter of this bacterial suspension was used to inoculate each *P. vulgaris* wild-type plant and plant with transgenic roots. To generate transgenic roots in *P. vulgaris* plants, the *Agrobacterium rhizogenes* K599 strain was used. The *A. rhizogenes* cells were cultivated on Luria-Bertani (LB) plates at 30°C for 2 days, with 100 μg/ml spectinomycin added to select plasmid vector.

### Plasmid construction

To investigate the promoter activity of the *PvNIN* (Phvul.009G115800), *PvTML* (Phvul.001G094400), and *PvPHR-L7*, 1500 and 1700 bp DNA fragments containing P1BS *cis*-regulatory elements were PCR amplified using specific primers, respectively (Table S3). The resulting fragments were cloned into the pENTR-D-TOPO vector (Thermo Fisher Scientific) and then recombined into the pKGWFS7 binary vector, which contains the GUS CDS. This generated transcriptional fusions, namely *pPvNIN:GUS* and *pPvTML:GUS*. To silence the expression of *PvPHR-L7*, a previously generated RNAi construct was used (Isidra-Arellano et al. 2020). Additionally, an overexpression construct of *PvPHR-L7* (Phvul.006G134700) was developed by amplifying the coding sequences (CDS) of 984 bp from the start codon ATG using gene-specific primers (Table S3). The amplified fragment was then cloned into the pENTR-D-TOPO vector (Thermo Fisher Scientific), and the resulting pENTR-*PvPHR-L7* plasmid was recombined into the pBA-DC-TdTomato binary vector to overexpress *PvPHR-L7* CDS under the 35S promoter. The resulting construct constitutively expresses the fluorescent Tandem-Double-Tomato (TdTomato) protein.

### *A. rhizogenes*-mediated transformation

Binary vectors with *pPvNIN:GUS* and *pPvTML:GUS*, *PvPHR-L7-RNAi*, or *PvPHR-L7*-overexpression constructs were individually mobilized into *A. rhizogenes* K599 by electroporation. The empty vectors pKGWFS7 or PBA-DC-TdTomato were used as controls. *A. rhizogenes*-mediated hairy root transformation was conducted according to the protocol described by Estrada-Navarrete et al. (2007). The resulting *P. vulgaris* plants, with transgenic roots and an untransformed shoot system, were grown in 2 l pots filled with wet vermiculite. Transgenic roots were examined for TdTomato fluorescence using a fluorescence stereomicroscope.

### Nodulation assays in *P. vulgaris* plants with transgenic roots

*P. vulgaris* plants with transgenic roots expressing the empty vector, *PvPHR-L7-*Overexpression*,* or *PvPHR-L7*-RNAi construct were transferred to 2 l pots containing wet perlite and inoculated with 1 ml of *R. tropici* (OD_600nm_ = 0.3). Rhizobia-inoculated plants were watered with a low-nitrogen (0.385 mM) Summerfield nutrient solution supplemented with 1 mM Pi (OPi conditions) or 5 μM Pi (LPi conditions). Inoculated plants were kept in a growth chamber at 25°C–27°C. Twenty-eight days after rhizobial inoculation, nitrogen-fixing nodules (mature nodules) were counted only on plants whose root systems and nodules showed TdTomato fluorescence.

### Protein expression and electrophoretic mobility shift assays

The full-length coding sequence of the *PvPHR-L7* gene was amplified from cDNA and cloned into the pENTR-D-TOPO vector (Thermo Fisher Scientific, USA). The resulting pENTR-*PvPHR-L7* plasmid was recombined into the pDEST™17 vector (Thermofisher, USA), and the resulting vector was transformed into *Escherichia coli* strain BL21. The recombinant proteins were purified using a Fast His-Tagged Protein Purification kit following the manufacturer's instructions (Zymo Research, USA). The biotin-labeled probes of the P1BS-containing promoter fragments were generated by annealing the biotin-labeled oligonucleotide and its non-biotin-labeled complementary oligonucleotide. Electrophoretic mobility assays (EMSA) were performed according to the manufacturer’s instructions of the Light-Shift Chemiluminescent EMSA Kit (Thermo Scientific, USA). Components of EMSA binding reactions included 1× binding buffer, 50 ng/μl poly(dI·dC), 1 unit of protein extract, 0.1 mmol of biotin-labeled target DNA, and varying concentrations of unlabeled target DNA (5, 10, and 20 mmol). All oligonucleotides are listed in Table S3.

### Transient transactivation assays in *N. benthamiana*

The *Agrobacterium tumefaciens* GV3101-mediated transient expression assay was performed in *N. benthamiana* leaves. The plasmids containing *35S:PvPHR-L7-MYC*, and the different transcriptional fusions *pPvNIN:GUS* and *pPvTML:GUS* were transformed into *A. tumefaciens* GV3101 and then co-infiltrated into *N. benthamiana* leaves. After 48 h, the leaves were incubated in GUS histochemical staining buffer at 37°C for 4–6 h. Then, the leaves were washed five times with 75% ethanol and photographed using a digital camera. Ten biological replicates were included in this experiment.

### Transactivation assays in *P. vulgaris* transgenic roots

Transgenic roots were generated as we described above. To this end, *P. vulgaris* seedlings were co-infected with *35S:PvPHR-L7-MYC* and *pPvNIN:GUS* or *35S:PvPHR-L7-MYC* and *pPvTML:GUS*. Individual constructs were used to generate transgenic roots of *P. vulgaris*, which served as controls. Transactivation assays were undertaken under non-symbiotic and symbiotic conditions. For non-symbiotic conditions, plants with transgenic roots were watered with optimal-nitrogen (5 mM) Summerfield nutrient solution supplemented with 1 mM Pi (OPi conditions) or 5 μM Pi (Deficient-Pi conditions). For symbiotic conditions, plants with transgenic roots were inoculated with *R. tropici* (O.D._600nm_ = 0.3) and watered with a low-nitrogen (0.385 mM) Summerfield nutrient solution supplemented with 1 mM Pi (OPi conditions) or 5 μM Pi (Deficient-Pi conditions). After 2 weeks of growing under LPi conditions and without rhizobia or after 5 days post-inoculation with rhizobia, transgenic roots were collected and incubated in GUS histochemical staining buffer at 37°C for 4–6 h. Then, roots were washed with 75% ethanol five times and photographed using a digital camera. Ten biological replicates were included in this experiment.

### Preparation of messenger RNA-seq libraries and next-generation sequencing

Total RNA was isolated from 0.5 g of transgenic roots bearing young nodules, expressing an empty vector (Control), *PvPHR-L7-*RNAi, or the *PvPHR-L7*-OX construct, and growing under OPi or deficient-Pi conditions. Stranded messenger RNA-seq (mRNA-seq) libraries were generated from 1 μg of genomic DNA-free total RNA from each experimental condition and prepared using the TruSeq RNA Sample Prep kit (Illumina, San Diego, CA, USA) according to the manufacturer's instructions. Six biological replicates, each containing roots with young nodules, were generated for each experimental condition. Each biological replicate contained one transgenic root with young nodules, showing TdTomato fluorescence, from 20 independent plants with transgenic roots. Therefore, each biological replicate from each experimental condition contained 20 transgenic roots with young nodules. We selected three biological replicates that showed similar transcript levels of *PvPHR-L7*, as determined by RT-qPCR, for RNA-Seq analysis. The selected biological replicates for *PvPHR-L7*-RNAi displayed an average five-fold reduction, whereas *PvPHR-L7*-OX showed an average four-fold increase in the *PvPHR-L7* transcript levels compared to the empty vector. In total, eighteen libraries were sequenced on an Illumina NextSeq 500 platform using a 150-cycle sequencing kit and a configuration of paired-end reads with a 75-bp read length. Library construction and sequencing were performed by the Unidad Universitaria de Secuenciación Masiva y Bioinformática (Instituto de Biotecnología, UNAM, México).

### Mapping and processing messenger mRNA-seq reads

Adapter and contamination removal were carried out using in-house Perl scripts. Sequences were filtered based on quality (Q33, FASTQ Quality Filter v0.0.13, http://hannonlab.cshl.edu/fastx_toolkit/index.html). Approximately 20 million reads per sample were aligned to the *P. vulgaris* transcriptome (v2.1 from Phytozome v13) using Bowtie2 (v2.3.5) and with the recommended parameters to meet the input requirements of RNA-seq by Expectation Maximization (RSEM) analysis (Langmead and Salzberg 2012). Gene expression was calculated using the RSEM method (v1.3.3) and with the default parameters (Li and Dewey 2011). Significantly DEGs (adjusted *P*-value ≤ .01) were identified using DESeq2, part of the Integrated Differential Expression Analysis MultiEXperiment (IDEAMEX) platform (Jiménez-Jacinto et al. 2019), with RSEM expected counts and using a Wald test. GO term enrichment analysis was performed using AgriGO (v2.0) and default parameters (False discovery rate (FDR) cutoff = 0.05) (Tian et al. 2017).

### RNAseq data validation by RT-qPCR

We randomly selected nine genes to validate our RNAseq data by RT-qPCR. To this end, transgenic roots bearing young nodules expressing an empty vector (control) or the *PvPHR-L7*-RNAi or *PvPHR-L7*-OX constructs and growing under OPi or deficient Pi conditions and showing TdTomato fluorescence were immediately harvested in liquid nitrogen and stored at −80°C until use. Following the manufacturer's instructions, total RNA was isolated from transgenic roots bearing young nodules of five different plants with transgenic roots using the ZR Plant RNA miniprep kit (Zymo Research, USA). cDNAs were synthesized from 1 μg of genomic DNA-free total RNA and used to analyze gene expression by RT-qPCR, as previously described in Isidra-Arellano et al. (2020). Primer sequences are available in Table S3.

### Gene expression analysis

To analyze the expression of *GUS*, *PvNIN*, *PvTML*, and *PvPHR-L7*, *N. benthamiana* leaves and *P. vulgaris* transgenic roots co-expressing *35S:PvPHR-L7-MYC* and *pPvNIN:GUS*, *35S:PvPHR-L7-MYC* and *pPvTML:GUS* were harvested, immediately frozen in liquid nitrogen, and stored at −80°C. Individual constructs and an empty vector were used to generate *P. vulgaris* transgenic roots or infiltrate *N. benthamiana* leaves, which were used as controls. Following the manufacturer's instructions, the total RNA was isolated from leaves or transgenic roots from four biological replicates using the ZR Plant RNA miniprep kit (Zymo Research, USA). cDNAs were synthesized from 1 μg of genomic DNA-free total RNA and used to analyze gene expression by RT-qPCR, as we previously described in Isidra-Arellano et al. (2020). Primer sequences are available in Table S3.

### Expression level of endogenous PvNIN and PvTML in wild-type roots and nodules

To analyze the expression of endogenous *PvNIN* and *PvTML*, wild-type *P. vulgaris* plants were grown under OPi or LPi conditions and inoculated with *R. tropici* or mock inoculum. Rhizobia- or mock-inoculated roots were harvested after 5 days post-inoculation, immediately frozen in liquid nitrogen, and stored at −80°C. Similarly, 10-, 15-, and 21-day-old nodules were harvested, immediately frozen in liquid nitrogen, and stored at −80°C. Following the manufacturer's instructions, the total RNA was isolated from roots and nodules from four biological replicates using the ZR Plant RNA miniprep kit (Zymo Research, USA). cDNAs were synthesized from 1 μg of genomic DNA-free total RNA and used to analyze gene expression by RT-qPCR, as previously described in Isidra-Arellano et al. (2020). Primer sequences are available in Table S3.

### Phylogenetic analysis of PHR transcription factors in legumes

To investigate the evolutionary relationships among *PHL* genes in legumes, we performed a phylogenetic reconstruction based on the predicted peptide sequences from *L. japonicus* (Lj1.0v1), *G. max* (Wm82 ISU-01 v2.1), *M. truncatula* (Mt4.0v1), *P. vulgaris* (v2.1), and *A. thaliana* (TAIR10). The *A. thaliana PHR1* protein sequence (AtPHR1; AT4G28610) was used as a query in BLASTP searches against the Phytozome v13 database (Goodstein et al. 2012) for each target species. All *A. thaliana* proteins showing significant similarity to AtPHR1 (*E*-value < 1e−10) were also retrieved to include other potential paralogs. Candidate homologs were retained if they exhibited significant similarity to AtPHR1 and contained a complete MYB DNA-binding domain, as determined using InterProScan v5.59 (Jones et al. 2014). The resulting peptide sequences were aligned using MAFFT v7.475 (Katoh and Standley 2013) under the Neighbor-Joining method with 100 bootstrap replicates to assess branch support. The resulting phylogenetic tree was visualized and annotated using the Interactive Tree of Life (iTOL) v6 (Letunic and Bork 2024).

### Statistical analyses and graphics

Statistical analyses and graphic generation were conducted using R software 4.1.2. The specific tests performed are indicated in the legend corresponding to them.

## Supplementary Material

pcp-2024-e-00206-File007_pcaf069

SupplementaryTable1_R3_pcaf069

SuppTableS2_R3_pcaf069

## Data Availability

The original contributions presented in the study are publicly available. The RNA-seq data presented in this study are deposited in the SRA database, accession number PRJNA1049907. https://www.ncbi.nlm.nih.gov/bioproject/PRJNA1049907
