# Supplementary material for: Phosphate deficiency reduces nodule formation through a phosphate starvation response-like protein in Phaseolus vulgaris
Source: Plant Cell Physiol. 2025 Jun 26;66(12):1794–810. doi: 10.1093/pcp/pcaf069 (PMC12739106; doi:10.1093/pcp/pcaf069)
Supplement: pcp-2024-e-00206-File007_pcaf069 [file pcp-2024-e-00206-file007_pcaf069.pdf]

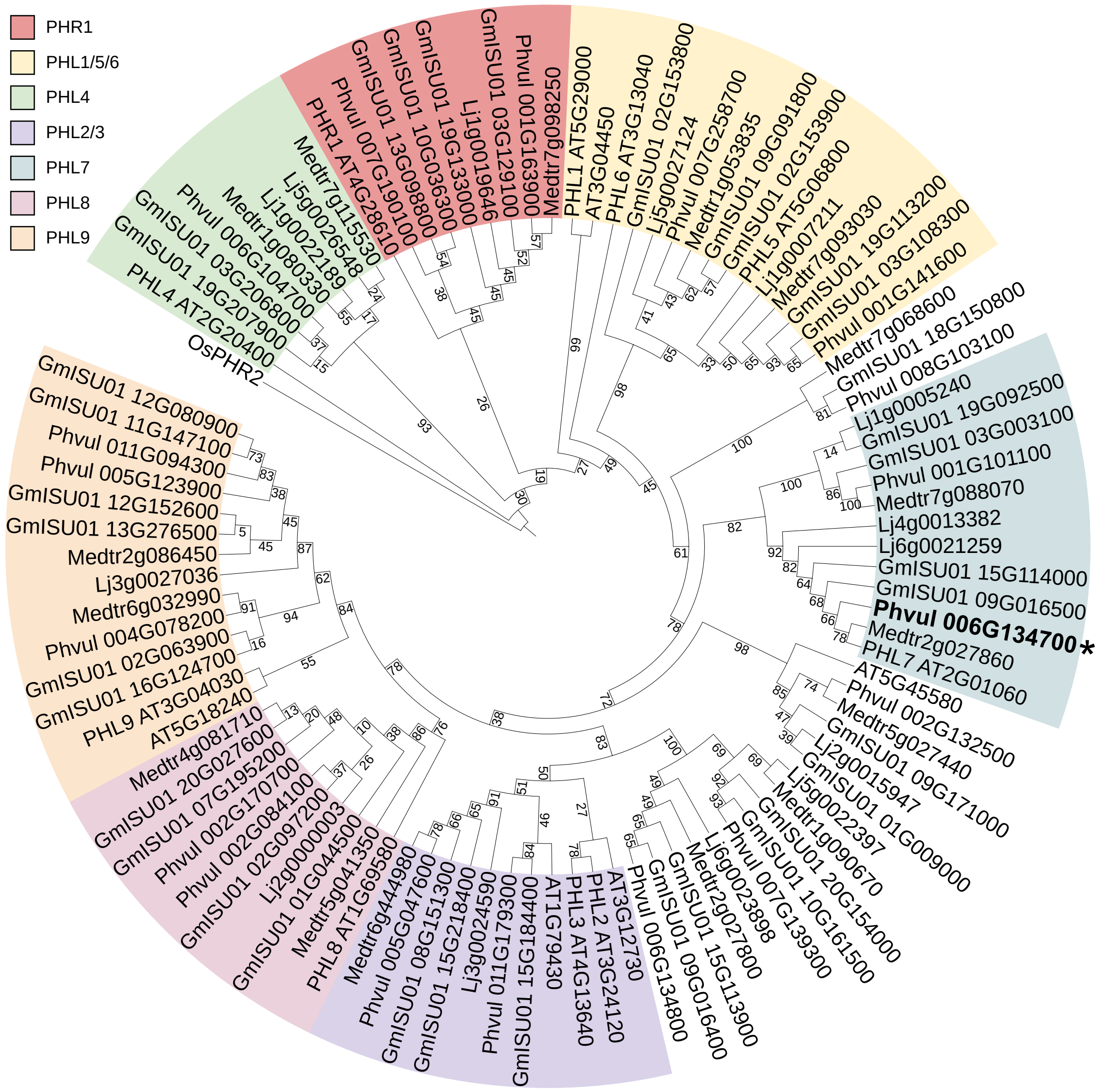

Figure S1. PvPHR-L7 belongs to the PHR transcription family. (A) To investigate the evolutionary relationships among *PHR1*-like genes in legumes, we performed a phylogenetic reconstruction based on the predicted peptide sequences from *Lotus japonicus*, *Glycine max*, *Medicago truncatula*, *Phaseolus vulgaris*, and *Arabidopsis thaliana*. The *A. thaliana PHR1* protein sequence (AtPHR1; AT4G28610) was used as a query in BLASTP searches against the Phytozome v13 database for each target species. Candidate homologs were retained if they exhibited significant similarity to AtPHR1 and contained a complete MYB DNA-binding domain, as determined using InterProScan v5.59. The resulting peptide sequences were aligned using MAFFT v7.475 under the Neighbor-Joining method with 100

bootstrap replicates to assess branch support. The resulting phylogenetic tree was visualized and annotated using the Interactive Tree of Life (iTOL) v6. According to this analysis, Phvul006G134700 groups into the PHR-L7 clade.

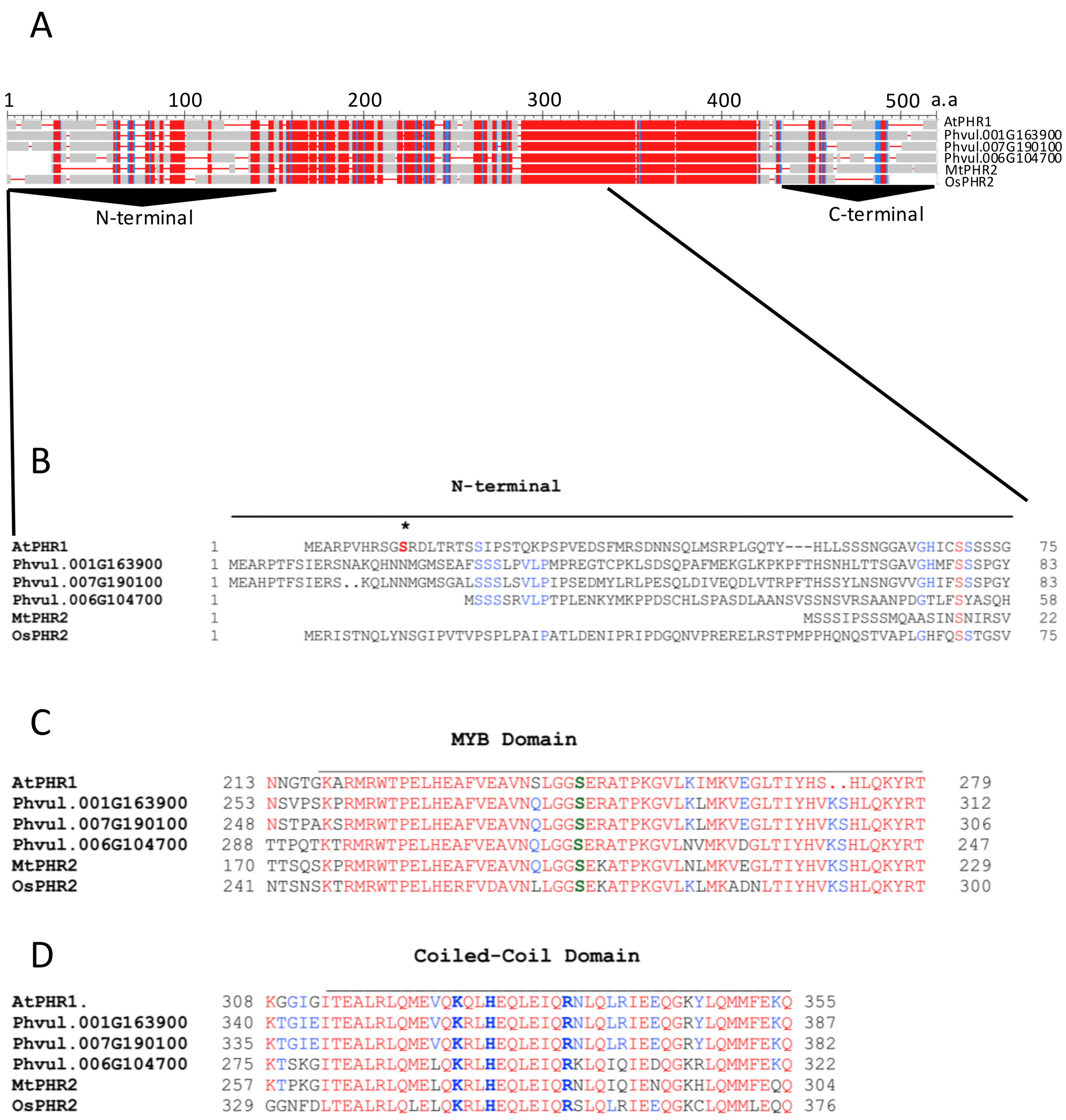

Figure S2. PvPHR-L7 possesses the characteristic domains and motifs of AtPHR1. (A) Diagram showing the amino acid sequences of *A. thaliana* PHR1 (AtPHR1), its two closed orthologous (Phvul.001G163900 and Phvul.007G190100) in *P. vulgaris*, and PvPHR-L7 (Phvul.006G104700) as well as PHR2, its functional orthologs in *Medicago truncatula* and *Oryza sativa*. The diagram was generated using the online service COBALT (https://www.ncbi.nlm.nih.gov/tools/cobalt/). (B) Amino acid sequence belonging to the N-terminal from AtPHR1, Phvul.001G163900, Phvul.007G190100, PvPHR-L7, MtPHR2, and OsPHR2. Serine highlighted in bold red font and

with an asterisk indicates the S11 residue reported to be phosphorylated by SnRK1 kinase in *A. thaliana* (Trejo-Fregoso et al. 2024). This serine residue is absent in PHR1 orthologs from *P. vulgaris*, *M. truncatula*, and *O. sativa*. (C) Amino acid sequence belonging to the Myeloblastoma (MYB) domain from AtPHR1, Phvul.001G163900, Phvul.007G190100, PvPHR-L7, MtPHR2, and OsPHR2. The serine highlighted in bold green is the residue phosphorylated by the GS3-SHAGGY-like kinase 2 (GSK2) in *O. sativa* and *A. thaliana* (Zhang et al. 2024). This serine residue is conserved in *P. vulgaris* and *M. truncatula*. (D) Amino acid sequence belonging to the Coiled-Coil domain from AtPHR1, Phvul.001G163900, Phvul.007G190100, PvPHR-L7, MtPHR2, and OsPHR2. Lysine (K), histidine (H), and arginine (R) highlighted in blue and bold are the residues where the SPX-Inositol Pyrophosphate8 binds to control PHR1 and PHR2 activity in *A. thaliana* and *O. sativa*, respectively. These amino acid residues are conserved in *P. vulgaris* and *M. truncatula* PHR proteins.

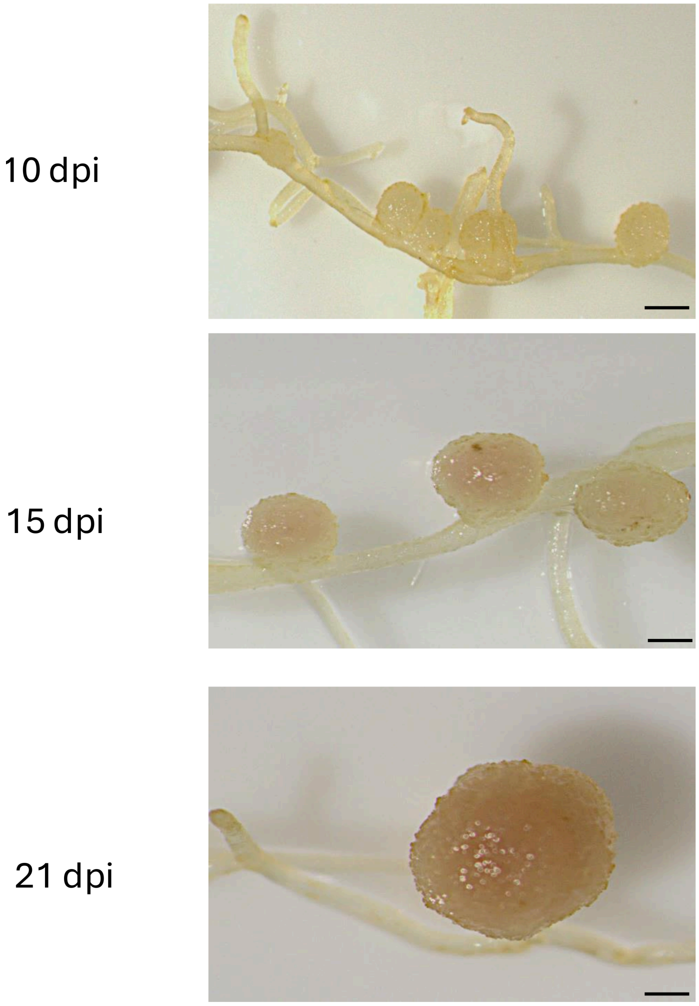

Fig. S3. ***Phaseolus vulgaris* roots bearing nodules at different developmental stages.** *P. vulgaris* plants were inoculated with *Rhizobium tropici* and, after ten days post-inoculation (dpi), developed small and white nodules. In contrast, *P. vulgaris* roots exhibit nitrogen-fixing nodules after 15 and 21 days post-inoculation with rhizobia. Bars represent 1mm.

A

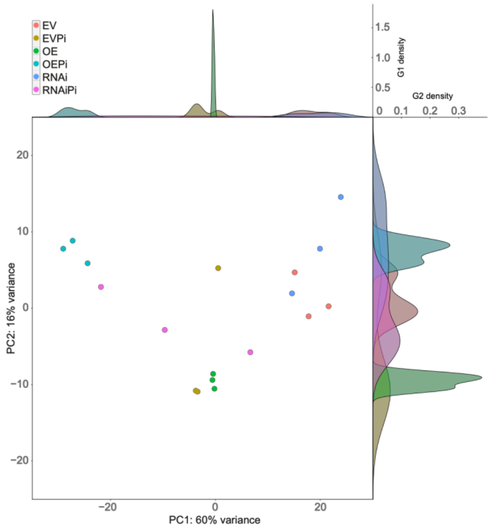

B

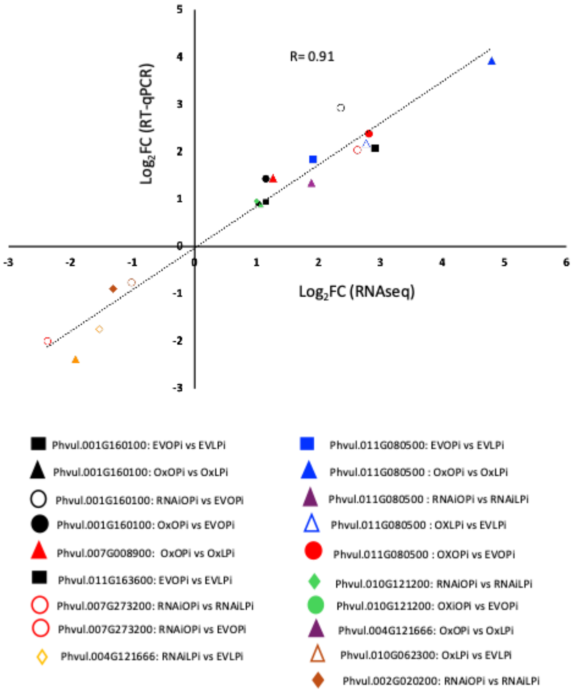

Figure S4. Principal Component Analysis (PCA) and RNA-seq validation by RT-qPCR. (A) PCA shows the variability of the RNA-seq data. (B) RNA-seq data validation by RT-qPCR. Nine genes were randomly selected to assess their expression levels. Each biological conditions contain three biological replicates. EV: Empty Vector. RNAi: *PvPHR-L7*-RNAi. Ox: *PvPHR-L7*-OX. OPi: Optimal Pi. LPi: Low Pi.

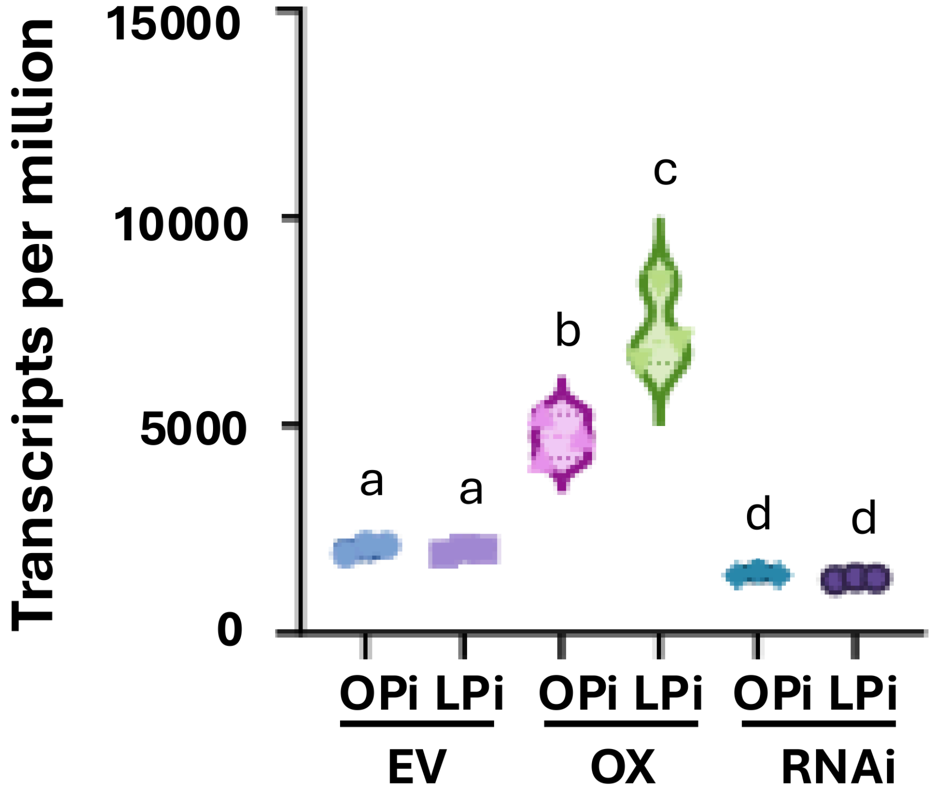

Figure S5. Transcript levels of *PvPHR-L7* in Empty vector, *PvPHR-L7*-RNAi, or *PvPHR-L7*-Ox roots bearing young nodules. *P. vulgaris* composite plants were grown under optimal Pi or low Pi conditions and inoculated with *R. tropici*. Three biological replicates containing roots with young nodules from 20 independent composite plants were included for each experimental condition. EV: Empty vector. OX: *PvPHR-L7*-OX. RNAi: *PvPHR-L7*-RNAi. OPi: Optimal Pi. LPi: Low Pi. Statistical classes sharing a letter are not significant.

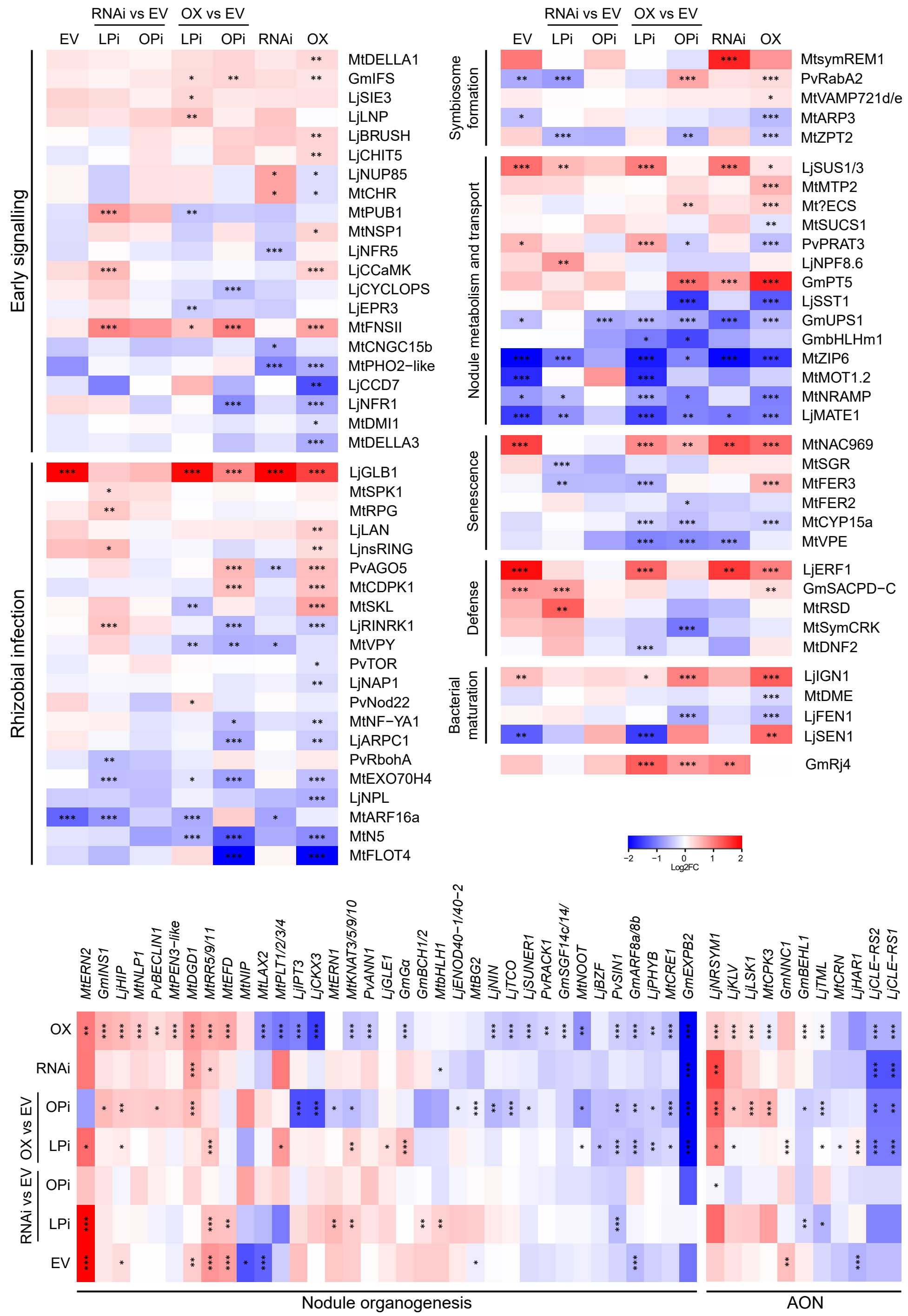

Figure S6. Modulation of the expression of *PvPHR-L7* affects the expression of symbiosis-related genes in *P. vulgaris*. Heatmap showing Log2 fold-change of root nodule symbiosis-related genes (Roy *et al*., 2020). Genes exhibiting higher and lower expression differences are displayed in shades of red and blue, respectively. EV: differentially regulated genes when comparing empty vector – optimal Pi vs. empty vector – low Pi conditions. RNAi

vs EV: differentially regulated genes when comparing *PvPHR-L7*-RNAi vs. empty vector under optimal Pi (OPi) or low Pi (LPi) conditions. Ox vs EV: differentially regulated genes when comparing *PvPHR-L7*-Ox vs. empty vector under OPi or LPi conditions. RNAi: differentially regulated genes when comparing *PvPHR-L7*-RNAi – OPi vs *PvPHR-L7*-RNAi – LPi (RNAi). Ox: differentially regulated genes when comparing *PvPHR-L7*-Ox – OPi vs *PvPHR-L7*-Ox – LPi (Ox) Asterisks indicate different levels of statistical significance of the comparisons (*: adjusted P-values<0.05; **= adjusted P-value<0.01; ***= adjusted P-value<0.001). Genes with no asterisk are not significantly differentially expressed.

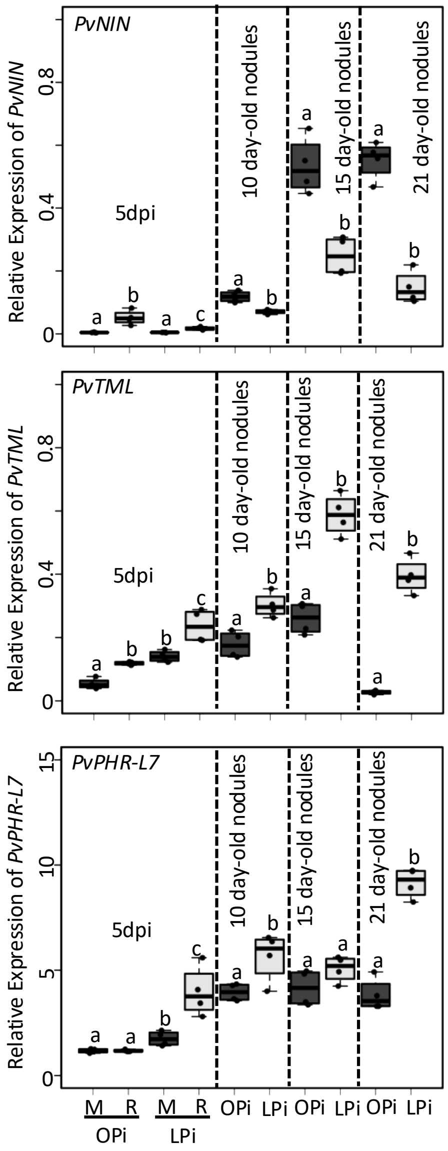

Figure S7. Pi deficiency affects the expression of *PvNIN*, *PvTML*, and *PvPHR-L7* across different stages of the root nodule symbiosis in *P. vulgaris*. The expression of *PvNIN* (A), *PvTML* (B), and *PvPHR-L7* (C) was evaluated by RT-qPCR. *P. vulgaris* wild-type plants were grown under optimal- (OPi) or low-Pi (LPi) conditions and inoculated with rhizobia. Mock (M) or rhizobia (R)-inoculated roots and roots bearing nodules were collected at different times after rhizobia inoculation. Box plots represent the first and third quartile (horizontal box sides) and the minimum and maximum (outside whiskers). Data were obtained from four biological replicates. One-way ANOVA followed by a Tukey honest significant difference (HSD) test was performed (P-value < 0.01). Statistical classes sharing a letter are not significantly different.

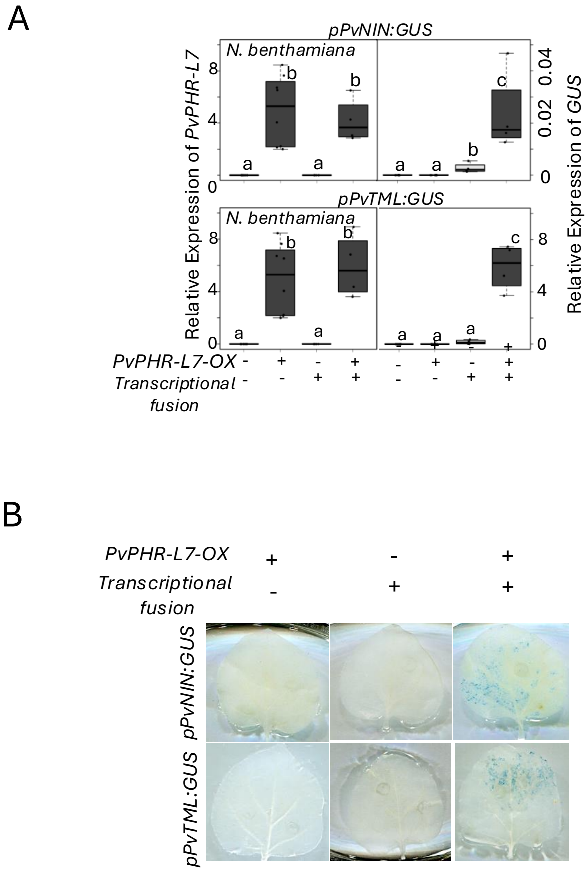

Figure S8. PvPHR-L7 transactivates the expression of *PvNIN* and *PvTML* in *Nicotiana benthamiana* leaves. Transactivation assays in *N. benthamiana* leaves were assessed by RT-qPCR (A) or by GUS activity (B). *pPvNIN:GUS* and *pPvTML:GUS* constructs alone or in combination with a *35S:PvPHR-L7-MYC* (*PvPHR-L7-OX*) construct were co-infiltrated in young leaves of *N. benthamiana* leaves. *N. benthamiana* leaves were assayed for *GUS* expression or activity two days after infiltration. Leaves were incubated in GUS histochemical staining buffer at 37°C for 4–6 h for *pPvNIN:GUS* activity, whereas 30 minutes for *pPvTML:GUS*. Images represent four biological replicates, each containing four leaves or twenty roots from independent plants. Box plots in panel A represent the first and third quartile (horizontal box sides) and the minimum and maximum (outside whiskers). Data were obtained from four biological replicates. One-way ANOVA followed by a Tukey honest significant difference (HSD) test was performed (P-value < 0.01). Statistical classes sharing a letter are not significantly different.

Supplementary Table S3: Primer sequences used for gene cloning or RT-qPCR analyses.

Primer Name	Sequence (5' – 3')
F-PvPHR-L7-OX	CAC CAT GTA TCA TTC AAA GAA TGT TCC TAG
R-PvPHR-L7-OX	TCA CAG ATT ACT GCC ACC AAC CT
F-promoterPvPHR-L7	CAC CGA CTA TAC AAA ATT TGT GTG TTT GA
R-promoterPvPHR-L7	TGA TTG GTT AGT GTG AGA AGG ATT
F-promoterPvNIN	CAC CAC TAA CAT GTT TCC AAA GAT TCC TTT
R-promoterPvNIN	TCC ATC ATT TTC AAA CTC TAG GTT
F-promoterPvTML	CAC CCA TAT TGC TCC TAA CAT TGA TCG
R-promoterPvTML	TTG CAG GCA AAT CTT CCT TCA ATA
Biotin-F-PromoterPvNIN	CAATTTCATTCTATAGCATATGCAACATTTTAGACAAAATCA
F-PromoterPvNIN (EMSA)	CAATTTCATTCTATAGCATATGCAACATTTTAGACAAAATCA
R-PromoterPvNIN (EMSA)	TGATTTTGTCTAAAATGTTGCATATGCTATAGAATGAAATTG
Biotin-F-PromoterPvTML	GAAATTAATATAAAAAGCATATACTAAAATTAAGCATATGTT
F-PromoterPvTML (EMSA)	GAAATTAATATAAAAAGCATATACTAAAATTAAGCATATGTT
R-PromoterPvTML (EMSA)	AACATATGCTTAATTTTAGTATATGCTTTTTATATTAATTTC
F-PvPHR-L7-Recombinant Protein	CAC CAT GTA TCA TTC AAA GAA TGT TCC TAG TG
R-PvPHR-L7-Recombinant Protein	GCA GAT TAC TGC CAC CAA CCT TAT CC
F- Phvul.010G121200	GCT GGC TCC TTC ATG ACA CTA
R- Phvul.010G121200	ACC CCT ATA TCA AAC TTG TCC CA
F-Phvul.001G160100	CGT TCA TGT GAA GGG GAT AAA
R-Phvul.001G160100	ATT CAA ACC GAT GTG ACA AGC
F- Phvul.011G080500	AAA GGG TCT GAA AGC TGC TGG
R- Phvul.011G080500	ATC AAC AGC CAC AAC CTC AGT
F-Phvul.007G008900	TGT CCA GTG CTC AGA GAA TGA
R-Phvul.007G008900	GAC CTC ATT TTC ACC TCA CGA
F- Phvul.004G121666	TCC ACA AGA AGC ACA GCA AAC
R- Phvul.004G121666	GCC TCT ACT CGT GTT CTA GCT
F-Phvul.011G163600	CCC TCT ATG GTT TTG GAG GTT
R-Phvul.011G163600	ACG GAG CAT AAC AAG GAT TCG
F- Phvul.010G062300	TAG GGT CAA TCA TCG CAT CCG
R- Phvul.010G062300	CGA CTC CTA AGC CTC CCA TTG
F-Phvul.007G273200	GGG GAG AAT GAT GGA AGT GAG G
R-Phvul.007G273200	AGC ACC CCT AAA TCA TGT AGC T
F- Phvul.002G020200	AGG TAG GCA TAT TCT GTG GTG G
R- Phvul.002G020200	ATA ATC TCC GGT GGC TGC ATG
F-PvPHR-L7 (RT-qPCR)	CAA GAC ATG GTG CTT CCA CTT
R-PvPHR-L7 (RT-qPCR)	TCT TGC CCA AGA AAA ACA GTG
F-GUS	ACACCGATACCATCAGCGATC
R-GUS	GTACCTTCTCTGCCGTTTCCA
F-PvNIN	CACCCATCATCCTCCCATTCC
R-PvNIN	TCACCCTAAAGCTACCACTGC
F-PvTML	AGGAGAGTGAAAATGGCAAGG
R-PvTML	GGGAAAAGATAGCACTTGAGGTT
F-PvRIC1	TATTGCGGACAGCCATCATCA
R-PvRIC1	TCCCTCTGGTGCGAGTCTAT
F-PvRIC2	CTCTCCATGCCCACACTCC
R-PvRIC2	TCATGTCCACGTTTTGCTAGG

References

Roy, S., Liu, W., Nandety, R.S., Crook, A., Mysore, K.S., Pislariu, C.I. *et al.* (2020) Celebrating 20 years of genetic discoveries in legume nodulation and symbiotic nitrogen fixation. *Plant Cell*. **32,** 15-41.

Trejo-Fregoso, R., Rodríguez, I., Ávila, A., Juárez-Díaz, J., Rodríguez-Sotres, R., Martínez-Barajas, E. & Coello, P. (2022) Phosphorylation of S11 in PHR1 negativelly controls its transcription activity. *Physiol Plant*. **174,** e13831.

Zhang, G., Wang, H., Ren, X., Xiao, Y., Liu, D., Meng, W. *et al*. (2024) Brassinosteroid-dependent phosphorylation of PHOSPHATE STARVATION RESPONSE2 reduces its DNA-binding ability in rice. *Plant Cell*. **36,** 2253-2271.
